# Anatomy of the Enigmatic Reptile *Elachistosuchus huenei* Janensch, 1949 (Reptilia: Diapsida) from the Upper Triassic of Germany and Its Relevance for the Origin of Sauria

**DOI:** 10.1371/journal.pone.0135114

**Published:** 2015-09-09

**Authors:** Gabriela Sobral, Hans-Dieter Sues, Johannes Müller

**Affiliations:** 1 Museum für Naturkunde Berlin, Leibniz-Institut für Evolutions- und Biodiversitätsforschung, Berlin, Germany; 2 Department of Paleobiology, National Museum of Natural History, Smithsonian Institution, MRC 121, Washington, DC, United States of America; National Cancer Institute, UNITED STATES

## Abstract

The holotype and only known specimen of the enigmatic small reptile *Elachistosuchus huenei* Janensch, 1949 from the Upper Triassic (Norian) Arnstadt Formation of Saxony-Anhalt (Germany) is redescribed using μCT scans of the material. This re-examination revealed new information on the morphology of this taxon, including previously unknown parts of the skeleton such as the palate, braincase, and shoulder girdle. *Elachistosuchus* is diagnosed especially by the presence of the posterolateral process of the frontal, the extension of the maxillary tooth row to the posterior margin of the orbit, the free posterior process of the jugal, and the notched anterior margin of the interclavicle. Phylogenetic analyses using two recently published character-taxon matrices recovered conflicting results for the phylogenetic position of *Elachistosuchus*–either as an archosauromorph, as a lepidosauromorph or as a more basal, non-saurian diapsid. These different placements highlight the need of a thorough revision of critical taxa and new character sets used for inferring neodiapsid relationships.

## Introduction

Since the beginning of the twentieth century, a brick-clay pit in the strata of the Arnstadt Formation has yielded a diverse assemblage of Late Triassic tetrapods. The locality lies along the present-day highway (Bundesstrasse) B79 between Halberstadt and Quedlinburg, on the southeastern edge of Halberstadt, in Saxony-Anhalt, Germany. The best-known taxon is the sauropodomorph dinosaur *Plateosaurus*, which is represented by some 50 specimens including at least two complete skeletons [1]. Otto Jaekel initially excavated the site between 1909 and 1912, and later Werner Janensch conducted additional excavations there between 1923 and 1928. Except for smaller digs by A. Hemprich in 1937 and 1938 no further work has taken place since the initial phase of exploration. The Late Triassic biota from Halberstadt includes bivalves, crustaceans, chondrichthyans, dipnoans, temnospondyls, stem-turtles, phytosaurs, and a haramiyid mammal [1–3]. The Arnstadt Formation is considered middle to late Norian in age based on biostratigraphic and cyclostratigraphic data [4].

Janensch [5] described and named a distinctive small reptile, *Elachistosuchus huenei*, based on remains found during the excavation of a skeleton of *Plateosaurus* around 1928. The holotype of *E*. *huenei* consists of six small blocks, which include a nearly complete but crushed skull, articulated vertebrae with associated humerus and ribs, and several vertebral and rib fragments. Aside from the holotype there is also unprepared material that was attributed to *Elachistosuchus*. Janensch [5] considered *Elachistosuchus* a pseudosuchian archosaur and diagnosed it by small body size, the lack of a specialized body plan, and the presence of a large posttemporal fenestra and an alleged antorbital fenestra. He explicitly compared this taxon to a variety of pseudosuchians.

Walker [6] reinterpreted *Elachistosuchus* as a rhynchocephalian closely related to the extant tuatara, *Sphenodon* based on the long anterior process of the jugal, the allegedly acrodont dentition, the large posttemporal fenestra, the absence of an external mandibular fenestra, and a strongly twisted end of the humerus. He also argued that the antorbital fenestra identified by Janensch [5] actually represented a damaged opening for the lacrimal canal. From his brief account it is not apparent whether Walker actually ever examined the original material.

The small size and fragility of the holotype of *Elachistosuchus huenei* do not permit additional mechanical preparation and thus no further studies were undertaken in recent decades. This led to this taxon to being largely ignored in the literature with the exception of occasional citations in connection with faunal reviews. Modern non-invasive techniques such as μCT scanning are often used for assessing internal anatomical information such as the structure of the braincase, inner ear, and cranial sinuses [7–9], but less for virtual preparation of them [10, 11]. The application of this technique has recently allowed for examination of the holotype and only known specimen of *E*. *huenei* and assessment of much of its internal cranial structure, helping to shed light onto controversial aspects of its anatomy. Furthermore, it also revealed previously unknown parts of the skeleton concealed in the matrix, specifically the braincase, palate, and parts of the shoulder girdle.

The objectives of the present study are to provide a more detailed description, including corrections of previous interpretations of the anatomy of *Elachistosuchus*, to analyze newly discovered skeletal elements, and to review its affinities using an a quantitative phylogenetic approach, with comments on the relationships of basal saurians.

## Materials & Methods

### Computed Tomography

**Fig 1:**
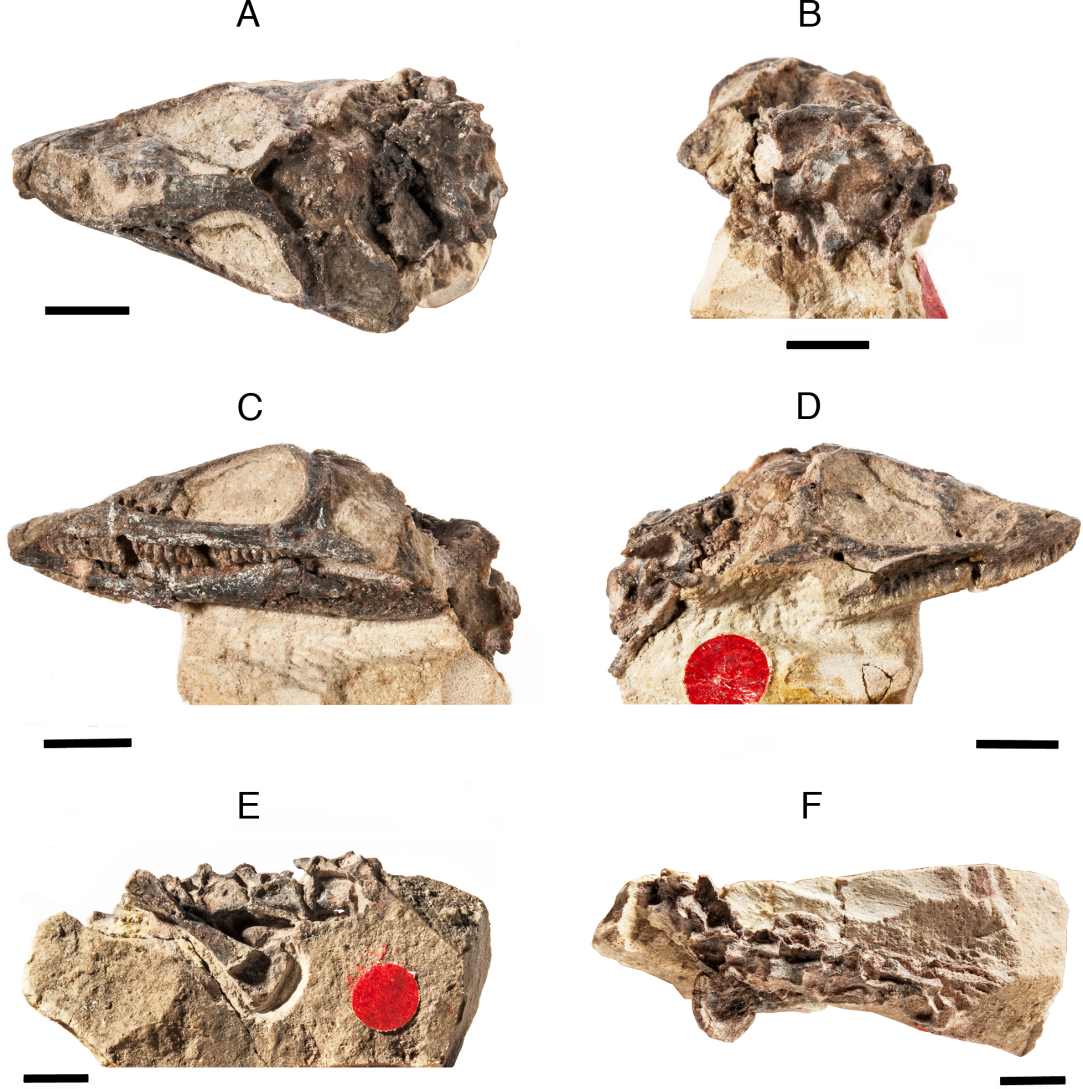
*Elachistosuchus huenei* MB.R. 4520 (holotype). Skull in A, left lateral and B, dorsal view. Articulated dorsal vertebrae and left humerus in C, lateral and D, dorsal view. Scale bars equal 1 cm.

The holotype of *Elachistosuchus huenei* comprises six blocks of bone-bearing rock, which are catalogued under a single number MB.R. 4520 (fossil reptile collection of the Museum für Naturkunde Berlin, Berlin, Germany). Janensch [5] numbered the individual blocks using the Roman numerals I–VI. Block I contains the skull (Fig 1A and 1B), block II articulated trunk vertebrae with associated humerus (Fig 1C and 1D), and blocks III to VI fragments of ribs, vertebrae, and gastralia. In the text, brief comparisons are made to *Euparkeria* (SAM-PK-7696—Iziko South African Museum, Cape Town, South Africa) and to *Prolacerta* (BPI/1/2675—Bernard Price Institute for Palaeontological Research, University of the Witwatersrand, Johannesburg, South Africa) based on work by GS.

The holotype of *Elachistosuchus huenei* was scanned at the Museum für Naturkunde Berlin using a Phoenix|x-ray Nanotom tomography machine (GE Sensing and Inspection Technologies GmbH, Wunstorf, Germany). Slices were reconstructed using the datos|x-reconstruction software, version 1.5.0.22 (GE Sensing and Inspection Technologies GmbH, Phoenix|x-ray) and the resulting volume was segmented and analyzed in VG Studio Max 2.1 (Volume Graphics, Heidelberg, Germany).

Settings for individual scans were as follows:

Block I: the piece in its entirety using 110 kV, 130μA, 1000ms, and voxel size 17.38 μm (two additional scans, one for details of tooth implantation and another for details of the braincase, both using 80 kV, 230 μA, 1000 ms, and voxel size of 9.84 μm).

Block II: 100 kV, 80 μA, 500 ms, and voxel size 38.33 μm.

Block III: 100 kV, 80 μA, 500 ms, and voxel size 39.99 μm (isolated material: a prepared vertebra lacking centrum scanned using 80 kV, 120 μA, 500 ms, and voxel size 8.49 μm; a small piece found lying close to this block using 60 kV, 240 μA, 750 ms, and voxel size 6.99 μm).

Block IV: 110 kV, 100 μA, 500 ms, and voxel size 55.55 μm.

Block V: 90 kV, 120 μA, 250 ms, and voxel size 37.99 μm.

Block VI: 80 kV, 200 μA, 250 ms, and voxel size 37.99 μm (broken-off piece 100 kV, 120 μA, 500 ms and voxel size of 14.29 mm).

### Phylogenetic Analysis

There are two recently published character-taxon matrices that cover basal diapsids and related taxa of reptiles: the dataset of Chen *et al*. [12] and that of Ezcurra *et al*. [13]. Chen *et al*. [12] constructed three different data sets for their analyses with different character treatments. In one of them, some of the characters were identified *a priori* as convergent aquatic adaptations found in all aquatic reptile taxa and scored as ambiguous in these taxa, i.e., coded as “?” (see [12] for details). The topology resulting from this dataset was pointed out as the preferred result for the authors, and thus this was the matrix used here to test for the phylogenetic relationships of *Elachistosuchus*. A full reassessment of taxa and characters for this purpose was beyond the scope of this study, and both previously published matrices were used without additional changes. *Elachistosuchus* was added in the matrices using Mesquite 2.75 build 564 [14] and analyzed using TNT 1.1 [15] with 1,000 replications, saving 20 trees per replicate. In the matrix of Chen *et al*. [12], characters were equally weighted and treated as unordered, but 36 characters were considered ordered in the matrix of Ezcurra et al. [13] (see original paper for details). For a better comparison with the Bayesian analysis, in which the characters were not ordered, an unordered analysis of the latter matrix was also performed. The coding of *Elachistosuchus* for each matrix can be found in the Supporting Information (S1 File). Consistency and retention indices were calculated with the STATS script downloadable in the TNT package. Bootstrap and jackknife values were calculated with 1,000 replicates and branches below 1 collapsed, the latter with 50% removal probability. Bremer support values were determined for suboptimal trees of up to 5 steps longer. Character optimizations were calculated with standard settings of the software.

In order to test the robustness of the phylogenetic relationships recovered, an additional Bayesian analysis was performed using MrBayes 3.2.1 [16], with all characters unordered. The analysis was run under implementation of the Mk model plus a gamma shape parameter (4 rate categories), 4 chains with a length of 4 million generations each, a sampling of every 100th generation, and a burn-in of 10,000. The "allcompat" consensus tree was used for summarizing the results. The average standard deviation of split frequencies was used to check for stationarity—i.e., if the value is <0.001, the analysis has reached convergence.

### Systematic Paleontology

Diapsida Osborn, 1903

Neodiapsida Benton, 1985

*Elachistosuchus* Janensch, 1949

Type species: *Elachistosuchus huenei* Janensch, 1949 (by monotypy)

*Elachistosuchus huenei* Janensch, 1949

#### Revised diagnosis

Small diapsid reptile with distinct posterolateral process of frontal, maxillary tooth row extending posterior to posterior margin of orbit, jugal with free posterior process, palatine ramus of pterygoid with shagreen of teeth, angular exposed along about one third of lateral surface of mandibular ramus, trunk ribs dichocephalous, notched anterior margin of interclavicle, posterior process of interclavicle spatulate.

#### Holotype

MB.R. 4520, partial skeleton comprising a nearly complete but crushed skull, articulated posterior cervical and anterior dorsal vertebrae with rib fragments, right humerus, bones of the shoulder girdle (interclavicle, clavicles, coracoids, and one scapula), and isolated dorsal vertebrae and rib fragments.

## Description

### Skull Roof

The anterior portion of the skull is badly damaged, and many bones are missing or incompletely preserved in this region.

The *premaxillae* are missing. Janensch [5] identified the right premaxilla as forming the anterior end of the upper jaw and noted that there are no sutures between the premaxilla and maxilla. The μCT images show some cracks in this area, but it is not possible to establish if any of these represent sutures. We thus consider the anterior ends of both upper jaws to be incomplete.

**Fig 2:**
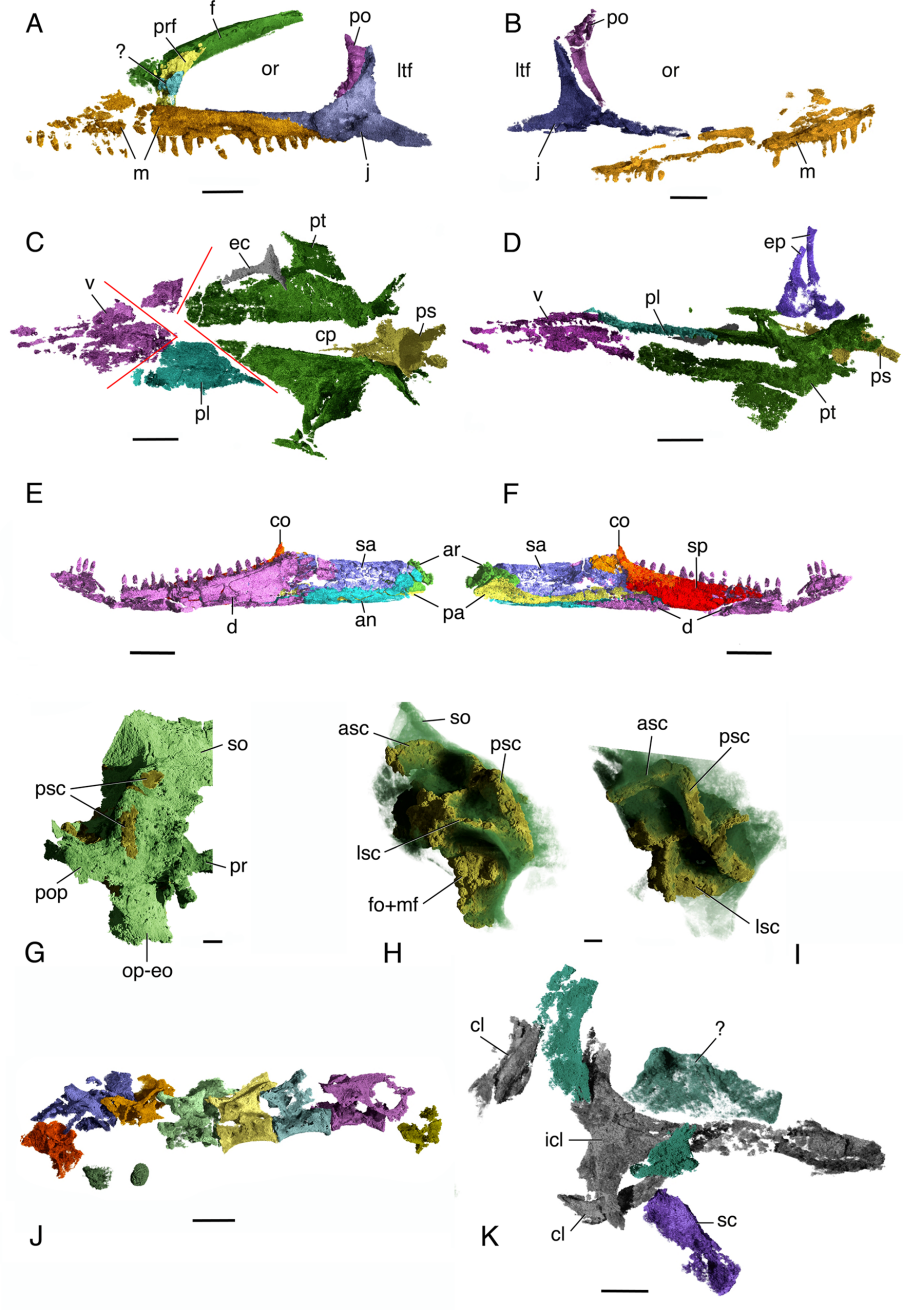
*Elachistosuchus huenei* MB.R. 4520 (holotype). A, B, segmented skull bones in A, left and B, right lateral views. Maxilla in orange, jugal in blue, ventral process of postorbital in purple, frontal-nasal complex in green, prefrontal in yellow, piece of bone that is here identified as a broken and displaced part of prefrontal. Scale bar equals 2 mm. C, D, segmented bones of the palate in C, dorsal and D, left lateral views. Vomers and anterior portions of palatine in purple, main body of left palatine in light blue, pterygoids and posterior part of palatines in green, ectopterygoid in gray, parabasisphenoid in yellow, and epipterygoid in dark blue. Scale bar equals 2.5 mm. Red lines indicate breaks along sutures between palatal elements. E, string of articulated trunk vertebrae in left lateral view. Scale bar equals 3 mm. F, G, left mandibular ramus in F, lateral and G, medial views. Coronoid in orange, dentary in purple, surangular in dark blue, angular in light blue, articular in green, prearticular in yellow, and splenial in red. Scale bar equals 2.5 mm. G-I, left side of the braincase in G, posterior and slightly lateral, H, left lateral and I, dorsal views. Braincase bones in green, inner ear structures in yellow. Scale bar equals 1 mm. J, segmentation of articulated trunk vertebrae (anterior to the left). K, ventral view of elements of the pectoral girdle. Interclavicle and clavicles in gray, scapula in purple, and unidentified elements in green. Scale bar equals 2.5 mm. Abbreviations: an, angular; ar, articular; asc, anterior semicircular canal; cl, clavicle; co, coronoid; d, dentary; ec, ectopterygoid; eo, exoccipital; ep, epipterygoid; cp, cultriform process; f, frontal; fo, fenestra ovalis; icl, interclavicle; j, jugal; m, maxilla; ltf, lower temporal fenestra; lsc, lateral semicircular canal; mf, metotic foramen; or, orbit; op, opisthotic; pa, prearticular; pl, palatine; po, postorbital; pop, paroccipital process; pr, prootic; prf, prefrontal; ps, parabasisphenoid; psc, posterior semicircular canal; pt, pterygoid; sa, surangular; sc, scapula; so, supraoccipital; sp, splenial; v, vomer.

As preserved, the *maxilla* (Fig 2A and 2B) bears at least 26 teeth although a total count of at least 30 is probable. The tooth row extends far posteriorly and ventral to the main body of the jugal. A distinct posterior extension of the maxilla and of the maxillary tooth row is found in basal neodiapsids such as *Youngina* [17] and in basal synapsids such as *Mycterosaurus* [18], and *Varanosaurus* [19], as well as some basal archosauriforms like *Proterosuchus* [20]. The conical tooth crowns curve slightly posteriorly. Anteriorly, the crowns are slightly flattened labiolingually, but, more posteriorly, they are circular in transverse section. Contrary to what was reported by Janensch [5], the tooth crowns do not bear vertical striations close to their apices. Tooth implantation is subthecodont as μCT images show that the teeth sit in shallow sockets (but see below). The maxilla is long and tapers posteriorly at its contact with the jugal. Although the dorsal processes of both maxillae incomplete, the one on the right side of the skull seems better preserved. It is not possible to estimate its full height. The process has a narrow base, extending for slightly less than a third of the total length of the maxilla. It likely formed the posteroventral border of the external naris, which faced more laterally than dorsally. The maxilla forms much of the ventral margin of the orbit. It also forms the anterior rim of the posterior entrance of the lacrimal canal.

**Fig 3:**
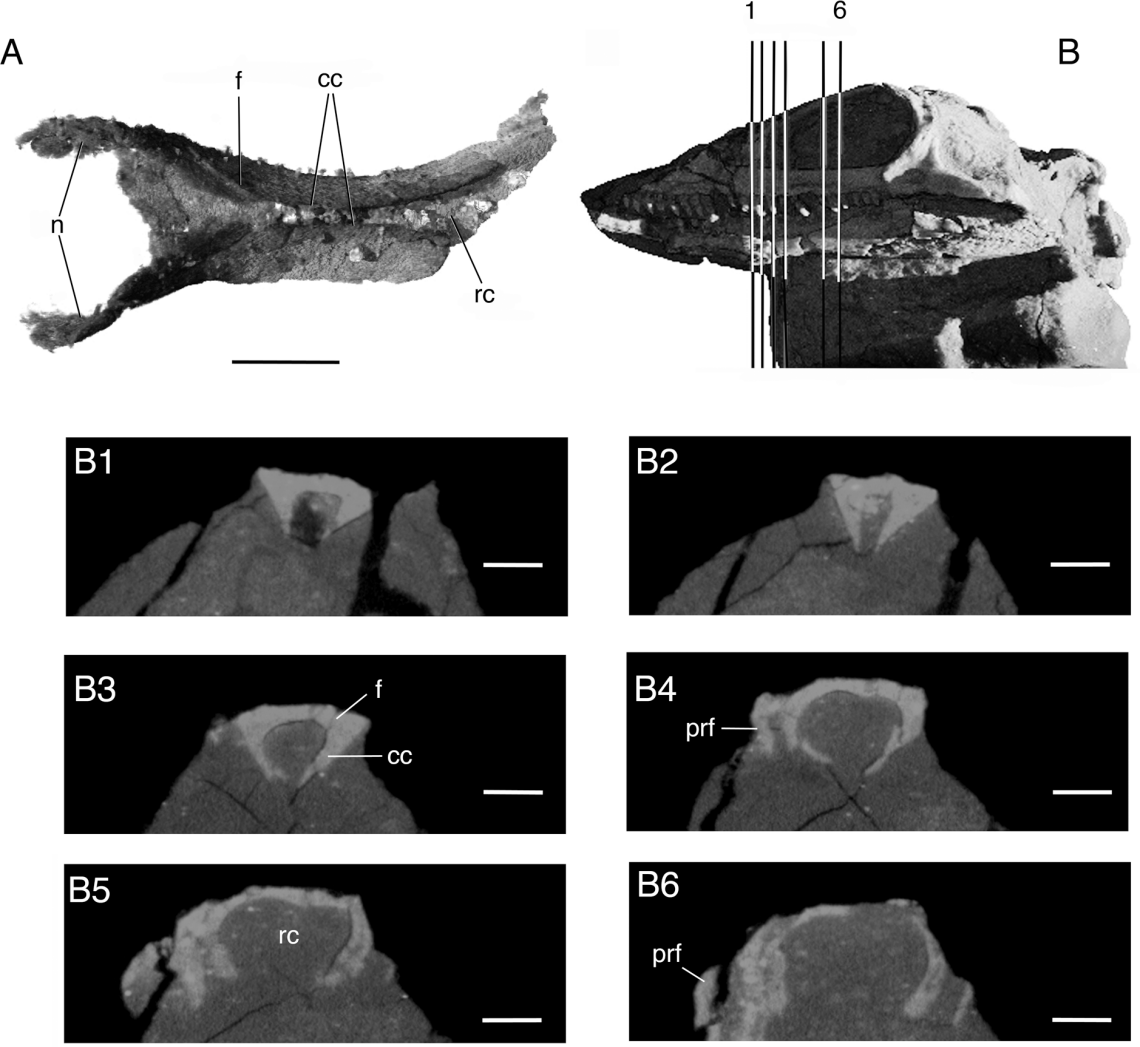
*Elachistosuchus huenei* MB.R. 4520 (holotype). A, fused frontals and nasals in ventral view. Scale bar equals 2 mm. B, left lateral view of the skull indicating a succession of cross-sections of the fused complex and the olfactory chamber (1–H). Scale bar equals 0.9 mm. Abbreviations: cc, crista cranii; f, frontal; n, nasal; prf, prefrontal; rc, rhinencephalic chamber.

Only the posteriormost portions of the *nasals* (Figs 2A and 3A) are preserved on both sides of the skull, in the region of their sutural contacts with the frontals. Thus, their anterior extent cannot be determined. Parts of the sutural lines can be seen in some individual slices, but it is not possible to trace them fully. For this reason, it is not possible to separately segment them and they are represented as a single complex in Fig 3. On the dorsal surface of the complex, an anteriorly concave line can be seen very clearly and almost uninterruptedly close to the anteriormost limit of the complex. Ventrally, a strongly sinuous line can also be seen close to the anteriormost extent of the complex forming sharp apices that meet in the midline. Due to their symmetrical nature on both left and right sides, we do not consider this to be an artefact. These features are identified as the dorsal and ventral contacts of the nasals with the frontals. The nasals are overlapped by the frontals, and thus their dorsal contact is more anteriorly positioned than their ventral one. Ventrally, the nasal forms the anterior part of the olfactory or rhinencephalic chamber [21].

There is no unambiguous trace of a *lacrimal*. We interpret an isolated piece of bone on the left side of the skull visible in the scans as a broken and displaced piece of the prefrontal (Fig 2A). The lacrimal is often represented by a very small, splint-like bone, which can be easily damaged or lost in fossil specimens (*e*.*g*., *Gephyrosaurus* [22]).

The *prefrontal* (Fig 2A) is partially preserved on the right side as a small triangular piece of bone and as a slightly larger one on the left side, but both bones are incomplete. The prefrontal forms the anterior border of the orbit. Its posterodorsal process does not contact the postfrontal. The prefrontal forms most of the posterior entrance of the lacrimal canal, except for the anterior rim formed by the maxilla. This confirms the interpretation by Walker [6] who first rejected Janensch’s [5] identification of this opening as an antorbital fenestra.

The *frontals* (Figs 2A and 3A) in *Elachistosuchus* are long, thick, and tightly sutured to each other along their straight median contact. The frontal participates extensively in the orbital margin, forming most of its dorsal rim. In dorsal view, the bone closely resembles those of *Pamelina* and *Kuehneosaurus* [23] in the presence of a posterolaterally directed posterior process, which contacts the parietal. The frontal of *Pamelina* has a posterior process that is nearly as long as the length of the main body of the bone, but this process is more distinct and slender in *Elachistosuchus*, as in *Kuehneosaurus*. The ventral surface of the frontal bears a prominent subolfactory process or *crista cranii* (Fig 3). It starts posteriorly on each bone as a short median ridge, which becomes deeper anteriorly and points only slightly medially, towards the crista on the opposite frontal. At about its posterior third, its base becomes thinner and the crista more concave and pronounced, so that, in the mid-portion of the frontals, the left and right cristae almost meet along the mid-line. At this point, they form a trough-like structure, with a subtle ridge extending along the median suture between the frontals. In the anterior third of the frontal, at the level of the anterior margin of the orbit, the crista sharply extends ventrally, then laterally, and finally fades out. The suture between the frontals and nasals is placed at about the level where the crista changes its direction. In lepidosauromorphs, the space enclosed by the cristae is occupied by the olfactory tracts and is termed the olfactory chamber (rhinencephalic chamber [21]). This structure of the frontal is very different from the condition in archosauromorphs, where the cristae cranii are generally much more subtle. They resemble more closely the cristae in lepidosauromorphs, as in the case of *Kuehneosaurus* and *Sophineta* [24], although they can be also quite shallow in taxa such as *Pamelina* [23] or *Gephyrosaurus* [22]. The cristae cranii of *Elachistosuchus* are similar to those of *Sophineta* but more sinuous, deeper, and more medially directed. Williston [21] considered long descending processes “primitive” and characteristic of the “early reptiles,” but there are still too few data on the three-dimensional ventral structure of neodiapsid frontals to permit rigorous phylogenetic evaluation.

The *postfrontal* is badly crushed on the left side and missing on the right one. It is not possible to assess its shape but it was fairly small and probably restricted to a small portion of the posterodorsal margin of the orbit.

The *postorbital* (Fig 2A and 2B) forms the posterior margin of the orbit. The anterior rim of the ventral process of the postorbital is strongly concave. The process is slender and tapers uniformly along its sigmoidal contact with the jugal. The posterior process of the postorbital is not preserved and thus its contribution to the upper temporal bar cannot be determined.

The three processes of the *jugal* (Fig 2A and 2B) extend almost perpendicular to each other. The anterior process is the most slender and longest of the three, almost reaching the anterior margin of the orbit. It forms most of the ventral rim of the orbit medially. Except for a short segment, it is almost completely concealed by the maxilla in lateral view. An elongate and gracile anterior process of the jugal is present in some rhynchocephalians (e.g., *Clevosaurus* [25]) but also in basal diapsids such as *Lanthanolania* [26]. The dorsal process of the jugal is long and forms the posteroventral margin of the orbit. The posterior process is subequal in length to the dorsal one but is more robust. It differs significantly from the jugal in basal lepidosauromorphs, where the posterior process is either smaller (*Gephyrosaurus*, *Pamelina*) or almost or altogether absent (*Kuehneosaurus*, *Sophineta*). In *Clevosaurus*, the posterior process of the jugal is longer and probably reached the suspensorium [25]. A long posterior process is present in *Sphenodon* and in basal diapsids such as *Orovenator* [27]. The lower temporal bar of *Elachistosuchus* was clearly incomplete because the posterior process of the jugal is complete and does not makes contact with any neighboring bone. The main body of the jugal is thick and bears a rounded depression on its lateral surface, which is more pronounced on the left element.

The temporal region of the skull of *Elachistosuchus* is badly damaged, and unfortunately none of the cranial elements in this region such as the *squamosals*, *quadrates* (but see below), and *quadratojugals* is preserved. There are also no traces of *postparietals* and *supratemporals*.

**Fig 4:**
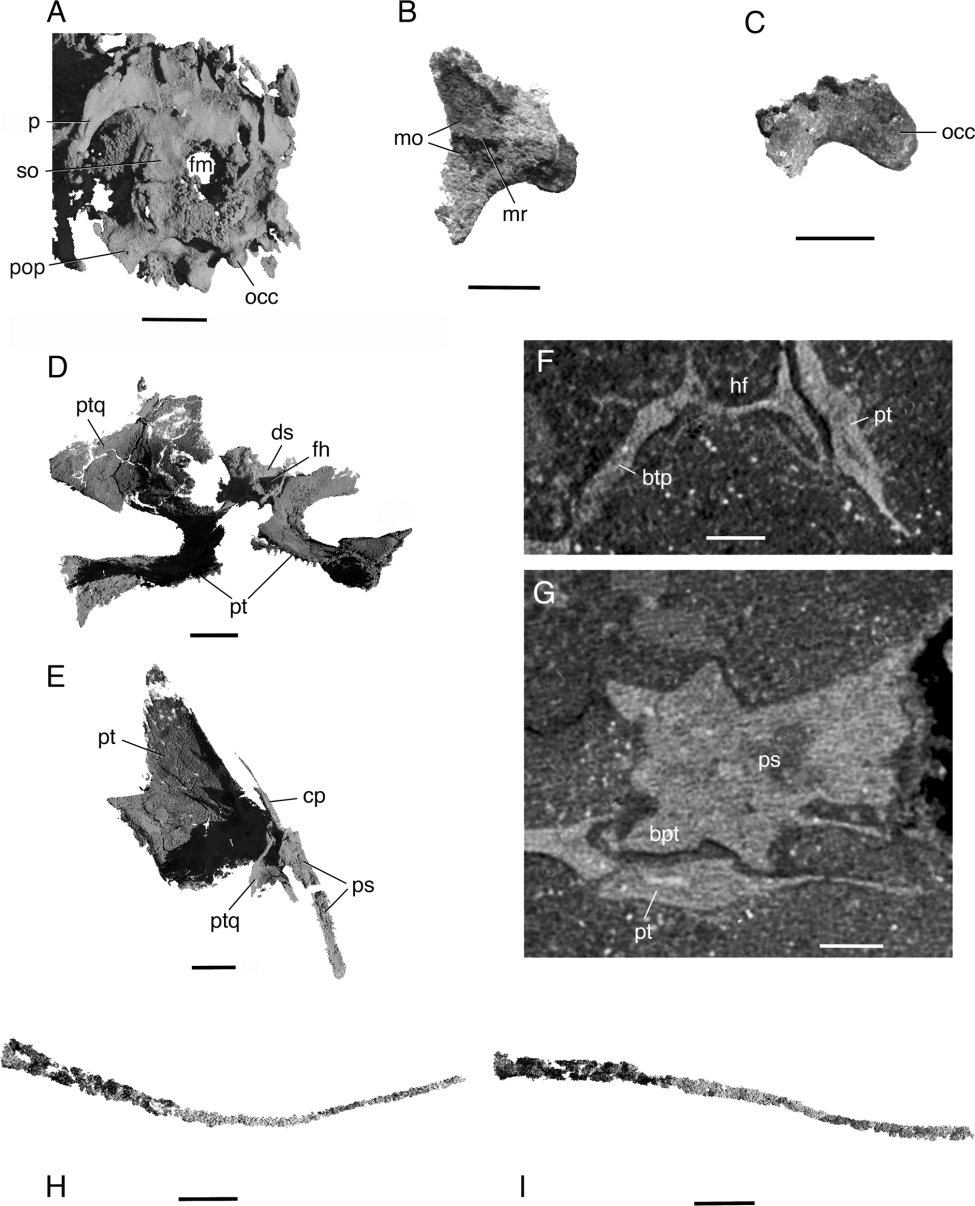
*Elachistosuchus huenei* MB.R. 4520 (holotype). Braincase and palate. A, occipital region in posterolaterodorsal view. Scale bar equals 2 mm. B, C, basioccipital in B, dorsal and C, left lateral views. Scale bar equals 1 mm. D, E, palate in anterior (showing the quadrate wings of the pterygoids and possibly part of the right quadrate) and posterolaterodorsal views (showing the basicranial articulation). Scale bar equals 1.5mm. F, G, details of the basicranial articulation in coronal and transverse sections. Scale bars equal 0.55 mm and 0.7 mm respectively. H, I, ceratobranchial in dorsal and lateral views. Scale bar equals 1.5 mm. Abbreviations: btp, basipterygoid process; cp, cultriform process; ds, dorsum sellae; fm, foramen magnum; hf, hypophyseal fossa; mo, surface for medulla oblongata; mr, median ridge; occ, occipital condyle; p, parietal; pop, paroccipital process; ps, parabasisphenoid; pt, pterygoid; ptq, quadrate wing of the pterygoid; so, supraoccipital.

The left *parietal* (Fig 4A) has been displaced posteriorly, and only its more posterior portion is preserved. The posterior border of the parietal is gently concave and forms a well-developed process that is not as strongly directed posteriorly as in *Gephyrosaurus* or *Youngina*, but more closely resembles those in *Pamelina*, *Tanystropheus*, and *Diphydontosaurus* [28]. Although only a small part of the right parietal is preserved, enough is visible to establish that the parietals were not fused to each other. The median contact between the bones is straight posteriorly.

### Palate

The posterior part of the palate was displaced ventrally, especially the lateral portion of the right side. The contacts between the bones of the palate are difficult to be identified with certainty. However, the palate is broken so that two pairs of lines were identified to likely represent these contacts (Fig 2C). We regard them as suture lines rather than breaks (or, possibly, as breaks along suture lines) because each pair is symmetrical on the left and right sides of the palate. The first pair is situated posteriorly, forming a ‘V’ with an anteriorly pointing apex. It seems to indicate the contact between the *pterygoids* and the *palatines*, and is more clearly visible on the left side. The left pterygoid is mostly intact, but because of the displacement of the right element, its anterior part seems to have been enlarged laterally. We cannot exclude the possibility that it includes part of the posteriormost extent of the palatine laterally, but this does not change our interpretation of the overall arrangement of the palate (see below). The second pair of symmetrical lines also forms a ‘V’, but the apex now points posteriorly. In this interpretation, the palatine is restricted to the mid-portion of the palate and the right one is mostly absent (see below).

The *vomers* (Fig 2C and 2D) were not fused. They are preserved in close association, but the left and right elements do not contact each other and a straight line can be traced medially between them. They bear small denticles and are relatively large, occupying approximately one third of the total length of the palate. According to our interpretations above, the vomers likely prevented the palatines from meeting along the mid-line. The *choanae* are laterally positioned and restricted to the anterior portion of the palate. The premaxillae appear to have been excluded from the choanal margins because the anteriormost elements of the palate as preserved are the vomers. The choanae are bordered by the vomers anteromedially and by the maxillae laterally. Most likely the palatine participated in the formation of the posteromedial border of the choana, but poor preservation renders this interpretation ambiguous.

The *palatine* (Fig 2C and 2D) is a large, trapezoidal element with small teeth and occupies the central portion of the palate. The right palatine is largely missing. In Fig 2C, it was incorrectly segmented along with the vomers and is shown as a small purple piece. As indicated above, the contact between palatine and pterygoid extended anteromedial to posterolateral, and it might be that part of the right palatine was segmented along with the pterygoid. The left one has a straight medial margin. The absence of associated pieces that would represent the right palatine leads us to conclude that the elements were separated entirely by the interpterygoid vacuity. The contact with the vomers extends posteromedial to anterolateral. Based on our interpretation, the palatines excluded the pterygoids from contact with the vomers. The contact with the maxilla is narrow, as in the basal archosauromorphs *Prolacerta* [17] and *Pamelaria* [29] and the basal rhynchocephalians *Gephyrosaurus* [22]. The palatine formed the anteromedial border of the elongate, narrow suborbital fenestra. The palatine likely participated in the posteromedial border of the internal choana, but the exact extent of this participation cannot be determined.

The *pterygoid* (Figs 2C, 2D and 4D–4G) is well preserved on both sides of the palate. It bears a shagreen of teeth on its ventral surface with the exception of the transverse flange, unlike in *Kuehneosaurus* but similar to the condition in other derived diapsids [23]. It is uncertain whether the pterygoid teeth formed distinct rows. The palatal ramus of the pterygoid is triangular, long and narrow anteriorly, and wider posteriorly. The lateral border of the process is smoothly concave, forming the medial margin of the suborbital fenestra. Because the medial border of the left palatine forms a straight line, we regard the interpterygoid vacuity as extending at least up to this element. Therefore, the left and right pterygoids did not contact each other anteriorly. The interpterygoid vacuity may have extended even further anteriorly since the medial margins of both vomers are also intact. The transverse flange of the pterygoid is broad, edentulous, and almost reaches the medial surface of the maxilla. It contributes to the posterior and posteromedial rims of the suborbital fenestra. The left transverse flange has been shifted ventrally. The basicranial articulation consists of a socket for the contact with the parabasisphenoid on the medial surface of the quadrate ramus of the pterygoid. The tall quadrate ramus of the pterygoid flares posteriorly first and then strongly laterally, covering part of the lateral sides of the parabasisphenoid around the basipterygoid processes and the anterior portion of the basal tubera. The contact between both bones is extensive and firm, indicating an immobile basicranial articulation. It is possible that part of the median wing of the quadrate is present and preserved in contact with the lateral surface of the pterygoid, but the extent of this wing and its contact cannot be ascertained (Fig 4D–4G).

Only the right *ectopterygoid* (Fig 2C) is partially preserved. It is a small triradiate element without teeth, consisting of a shorter lateral head for contact with the maxilla and jugal and a more slender medial process for contact with the pterygoid. Both the dorsal and ventral surfaces of the lateral head bear ridges extending parallel to the long axis of the bone.

Both *epipterygoids* are preserved (Fig 2D). They are well developed and their ascending (dorsal) part consists of a stout, slightly posteriorly arched bar extending from the basicranial articulation to the skull roof. The broad ventral base of the epipterygoid is close to the parabasisphenoid, and it probably took part in the basicranial articulation in a manner similar to that in *Sphenodon*.

Medial and parallel to the posterior half of the lower jaw, there is a thin and long bone that is identified here as probably the left *ceratobranchial* (Fig 4H and 4I). The expanded anterior end of this element is flattened dorsoventrally and bears a small opening. From its midpoint back, the shaft of the ceratobranchial tapers posteriorly. In dorsal view, it is gently concave medially.

### Braincase and Inner Ear

Despite having been pushed posteriorly and ventrally during fossilization, as well as rotated slightly to the right, the braincase is well preserved, allowing for a detailed description of its elements.

The *parasphenoid* and *basisphenoid* (Figs 2C, 2D and 4D–4G) are preserved roughly in their position of contact with the palate, anterior to the braincase and detached from it. Both elements are tightly fused to each other and thus will be described together as *parabasisphenoid*. The parabasisphenoid is a large, pentaradiate structure with a long, slender cultriform process that is V-shaped in transverse section, with the apex of the outline oriented ventrally. It bears a large and elongate hypophyseal fossa (sella turcica), although the degree of elongation is likely exaggerated by the detachment of the compound bone from the braincase. The hypophyseal fossa is shallow, open anteriorly, and laterally bordered by short clinoid processes that become slightly taller posteriorly, comparable to the condition in *Youngina* [30]. The dorsum sellae is tall, considerably higher than the clinoid processes, and its lateral borders are directed posterolaterally. The basipterygoid processes (Fig 4D, 4F and 4G) are long and project ventrally. Each has a small head for the contact with the dorsal concave surface of the pterygoid. This structure differs from the apparently button-like structure in basal diapsids such as *Lanthanolania* or lepidosauromorphs such as *Kuehneosaurus* and *Gephyrosaurus*, but is more similar to the processes in *Youngina*, *Orovenator*, *Sphenodon*, and possibly *Prolacerta*. Posterior to the basipterygoid process, the parabasisphenoid expands considerably to form the anteromedial part of the braincase and contact the basioccipital. In transverse section, the posterior portion of the parabasisphenoid is smoothly concave, with slightly laterally directed lateral walls that distinctly decrease in height posteriorly. Unfortunately, the quality of preservation does not allow for the identification of structures such the vidian sulcus or the canal for passage of the internal carotid artery or grooves/foramina for the abducens nerve (CN VI).

The *basioccipital* (Fig 4B and 4C) can be readily identified in the scans. It is approximately 2.2 mm in length and contributed at least to the posterior part of the basal tubera. The poorly preserved occipital condyle is clearly set off from the basicranium by a well-defined neck, but it was relatively small compared to the diameter of the foramen magnum. The dorsal surface of the basioccipital bears two shallow concavities separated by a low median crest for the medulla oblongata.

The *exoccipitals* (Figs 2H–2J and 4A) are poorly preserved, and it proved impossible to segment them as distinct entities. However, it is also impossible to confirm if they were fused to the opisthotics. They appear in the scans as an indistinct mass around the foramen magnum. As noted above, it is not possible to establish whether they met on the dorsal and/or ventral margins of the foramen. They likely formed the posterior border of the metotic foramen.

Both *opisthotics* (Figs 2H–2J and 4A) are preserved in partial articulation with the other braincase elements, although they are displaced and badly crushed on both sides. The paroccipital process is prominent and was correctly identified by Janensch [5]. It is strongly flattened anteroposteriorly and slightly longer than wide, with a subtle constriction at about mid-length that separates its proximal end from its distal portion. The ventral margin of the paroccipital process is slightly posteriorly directed, as in many archosauromorphs such as *Euparkeria* (SAM-PK-7696) and *Prolacerta* (BPI/1/2675). Most of its ventral part is much damaged, making it impossible to provide a more detailed description of the lateral wall of the braincase.

Both *prootics* (Fig 2H–2J) are mostly crushed and displaced. Most of the lateral wall of the left prootic seems to have shifted medially, where one large, round foramen can be seen. The prootic participates posteriorly in the fenestra ovalis, forming its anterior border and in the trigeminal (CN V) foramen anteriorly, forming its posterior border. The main body of the prootic is perforated by the facial (CN VII) foramen, whereas the foramina for the anterior and posterior branches of the vestibulocochlear nerve (CN VIII) are only visible medially when the medial wall of the inner ear is well-ossified–which is not the case in *Elachistosuchus* (see below). The foramen of the CN VII is usually very small, whereas the one for the CN V is large. However, identifying this large, round foramen as the trigeminal opening is problematic. First, the foramen of the CN V is located anteriorly so that the prootic shows only a notch on its anterior border [31]. The ventral border of this foramen is formed by the anteroventral process of the prootic. In cases where a laterosphenoid is present, the anterior border of the trigeminal foramen is formed by that element [32]. A foramen fully enclosed by the prootic is only found in more derived archosaurs (e.g. *Dysalotosaurus*, [33]). Secondly, the anteroventral process of the prootic is present in *Elachistosuchus*, and the border dorsal to it is anteriorly concave. Thus, we identify this round foramen on the prootic as the facial foramen and regard its enlargement as an artefact of preservation. We also identify the prootic as having a well-developed anteroventral process and forming the posterior and ventral borders of the trigeminal foramen.

The *supraoccipital* (Figs 2G–2I and 4A) appears to have had little or no exposure on the dorsal surface of the skull, as in many basal neodiapsids and in *Sphenodon*. Close to its lateral left border, the supraoccipital bears an opening, which Janensch [5] interpreted as the posttemporal fenestra. Based on the μCT images, this opening actually represents the damaged posterodorsal wall of the osseous posterior semicircular canal. This opening is continued ventrally, on the left opisthotic/exoccipital, and a similar structure is present on the right side. At the middle of the opening, a bar of bone remains, and it seems to indicate the contact between the supraoccipital and opisthotic-exoccipital. The open posterodorsal wall of the posterior semicircular canal is then divided into dorsal and ventral parts. Taking this bar of bone as the contact between the aforementioned braincase elements, the supraoccipitals participated in the dorsal margin of the foramen magnum. Overall, the supraoccipital of *Elachistosuchus* is similar to that of *Prolacerta* [31].

The *inner ear* (Fig 2H–2J) of *Elachistosuchus* is mostly open medially as there is no trace of medial ossifications of the prootic and opisthotic in the CT images. This is likely not an artefact because this condition is also present in most diapsids and basal saurians such as *Youngina* [30, 34]. As explained above, the lateral configuration of the inner ear is less clear due to extensive damage in this region. Due to poor preservation of this area of the braincase, it is unclear whether the borders of the fenestra ovalis was less well-defined, as in *Youngina*, or if its borders were more ossified as in *Prolacerta* [31]. However, the preservation of the material allows for the description of the inner ear itself because an area of sediment with a density different from the rest of the matrix turned out to correspond to the impression of the soft structures of the inner ear. The inner ear is not completely preserved on either side. On the right side only the common crus is present, together with parts of the anterior and posterior semicircular canals. On the left side, the dorsal and posterior parts of the inner ear are preserved. Only the posterior part of the metotic foramen is preserved. This opening was apparently not subdivided. The anterior part of the vestibule and the cochlea are also not preserved. Only the posterior half of the anterior semicircular canal can be identified; the canal is directed posteromedially. The posterior semicircular canal leaves the vestibule on its posterior part, close to its medial border. In dorsal view, it describes a gentle curve that first extends anterolaterally and then medially to the common crus, which was enclosed by bone. The lateral semicircular canal is dorsal to the paroccipital process, and thus to the vestibule, a feature reported to be present in basal archosaurs [35]. However, as both *Elachistosuchus* and *Euparkeria* (GS pers. obs.) show this structure, it is possibly a plesiomorphy for more basal saurians, although the exact nature of this character is unclear. Posterior to the common crus, there is a communication between the inner ear and the cranial cavity by means of a foramen that is only dorsally delimited by bone. A similar structure is present in the braincase of *Youngina*, where it was identified as the opening of the endolymphatic duct [30]. This contrasts with the condition in derived lepidosaurs such as *Ctenosaura* where the opening of the duct lies slightly more anteriorly, dorsal to the posterior auditory foramen [36], and also differs from archosaurs, as exemplified by the ornithopod dinosaur *Dysalotosaurus*, where the duct opens more anteriorly and dorsal to the acoustic recess [33]. The medial wall of the braincase is mostly open; only the dorsal portions of the constituent bones are present, indicating an enlarged internal acoustic meatus. The *stapes* could not be identified.

**Fig 5:**
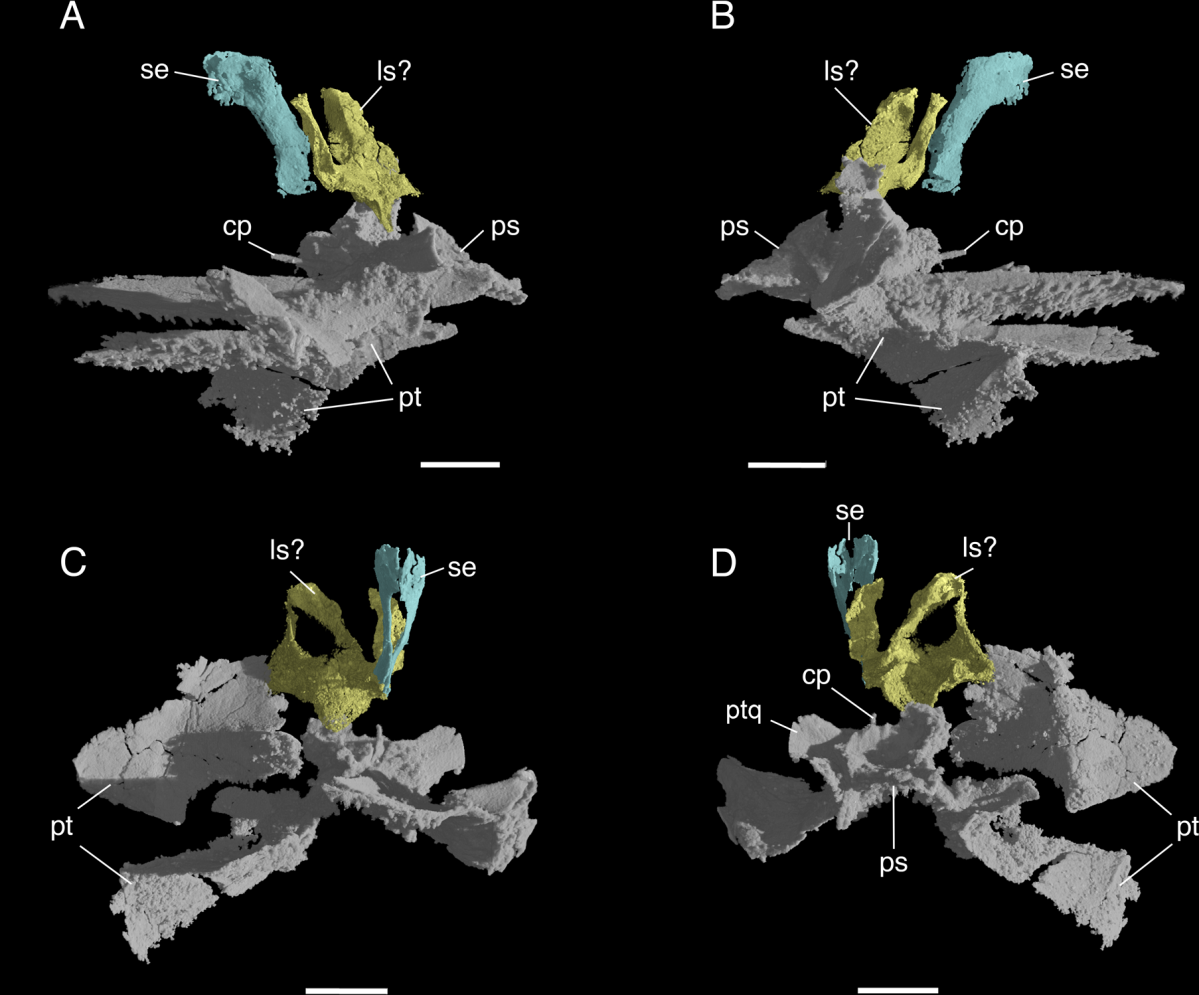
*Elachistosuchus huenei* MB.R. 4520 (holotype). Anterior braincase ossifications in A, left lateral, B, right lateral, C, anterior, and D, posterior views. Palatal and braincase elements in grey, sphenethmoid in green, and unidentified element in yellow. Scale bars equal 2 mm. Abbreviations: cp, cultriform process; ls, laterosphenoid; ps, parabasisphenoid; pt, pterygoid; ptq, quadrate wing of the pterygoid; se, sphenethmoid.

Two additional elements were found in the anterior region of the braincase that we identify as the ossified *sphenethmoid* and tentatively the right prootic with the *laterosphenoid*. Of these, the sphenethmoid is the better-preserved element. It is formed by two thin sheets of bone (left and right ones) located between, and slightly anterior to, the epipterygoids, and dorsal to the cultriform process of the parabasisphenoid. Dorsally, they reach up to the dorsal limit of the material, but are not in articulation with the skull roof because at the latter is not preserved in this region. Left and right counterparts descend ventrally from the midline and, posteriorly, these parts diverge laterally to form an anteriorly facing opening. Further posteriorly, they meet again in the midline and extend shortly ventrally. It is thus a roughly Y-shaped element whose parts forming the dorsal planum supraseptale meet dorsally along the midline and whose interorbital component is rather short. Situated posterior to the sphenethmoid, but not in contact with it, there is the second element, which is incompletely preserved (Fig 5). We tentatively identify this as the laterosphenoid. Its posterior extension on the right side seems to indicate that the anterior part of the right prootic might be part of it. It is, however, difficult to identify anatomical features characteristic of both bones.

### Lower Jaw

The right mandibular ramus is missing and the left one is badly damaged, especially at its anterior and posterior ends. Due to extensive fracturing the sutural separations between the elements are difficult to trace in several places and thus the outlines of individual bones (Fig 2E and 2F) must be considered tentative. Due to the poor preservation, identification of foramina or areas for muscle attachments is not possible. The ventral margin of the mandibular ramus is straight but the tooth-bearing dorsal margin of the dentary is gently concave, especially posteriorly. The overall configuration of the lower jaw is, to varying degrees, similar to that in basal archosauromorphs such as *Pamelaria* [29] and *Prolacerta* [17] but also resembles that in *Askeptosaurus* [37].

The *articular* and *prearticular* are tightly sutured to each other, but it is still possible to differentiate these two elements for most of the extension of their contact. The articular is restricted to the posterior end of the mandibular ramus. It is Y-shaped, with a long and slightly medially directed ventral component. The dorsal surface of the articular exclusively forms the glenoid fossa and is strongly concave in transverse section in its mid-point. The articular takes part in the modestly developed retroarticular process and also forms the posterior wall of the adductor fossa.

The *prearticular* is a long and thin bone that forms most of the posteromedial region of the lower jaw. It sheaths the medial surface of the articular and contributes to the dorsomedial wall of the adductor fossa. The prearticular extends anteriorly, contacting the splenial anterior to the coronoid process and at the level of the last dentary tooth. It describes a concave arc along its way, forming the dorsomedial margin of the adductor fossa. The adductor fossa faces dorsomedially and is deeply excavated medially, with a low medial wall. In this regard, *Elachistosuchus* is similar to *Prolacerta*.

The *angular* forms the posteroventral part of the medial surface of the mandibular ramus and the adductor fossa, extending anteriorly slightly further than the prearticular. The angular also had lateral exposure, but its exact extent is difficult to determine because the poor preservation of this part of the lower jaw does not permit confident tracing of the suture between the angular and surangular. The angular contributes to the retroarticular process.

The *surangular* forms the posterodorsal part of the lateral surface of the mandibular ramus, thus contributing to the lateral wall of the adductor fossa. The suture with the dentary is difficult to trace due to extensive fracturing in this region. It is possible that the break represents, to some extent, the contact between surangular and dentary. If this is the case, the surangular dominated the posterolateral region of the lower jaw, extending as far anteriorly as the sixth preserved dentary tooth (counting from the posterior end of the tooth row) and well anterior to the coronoid process. This configuration is similar to the one found on the lateral surface of the mandibular ramus of *Askeptosaurus* and, to a lesser extent, to that of *Tanystropheus* [38].

The anterior limit of the *coronoid* cannot be determined, but it was likely restricted to the coronoid process and is mostly visible in medial view, being concealed by the surangular laterally. The coronoid process is not greatly elevated; this region of the lower jaw is less well developed in *Prolacerta* and *Askeptosaurus*, and the mandibular ramus of *Elachistosuchus* resembles that of *Pamelaria* more closely in this respect.

The *splenial* forms most of the medial surface of the lower jaw and contacts the dentary at the midpoint of its ventral margin. Its extended dorsal margin conceals the medial exposition of the dentary and provides support for the lower teeth. Posteriorly, the splenial reaches the beginning of the coronoid region, and it extends very far anteriorly, covering most of the Meckelian canal but not reaching the mandibular symphysis and thus leaving the anterior end of the canal uncovered. The canal is distinct and occupies most of the medial surface of the lower jaw. The anterior extent of the splenial is pronounced in basal neodiapsid and saurian taxa, although the Meckelian canal does not reach as far anteriorly in *Askeptosaurus* as it does in *Elachistosuchus* or *Pamelaria*.

The *dentary* has 20 preserved teeth but may have had at least 25. The teeth are very similar to those of the maxilla, without any marked increase in size throughout the tooth row (see below). Posteriorly, the tooth row almost extends to the coronoid process but it is shorter than the maxillary tooth row.

### Dentition

**Fig 6:**
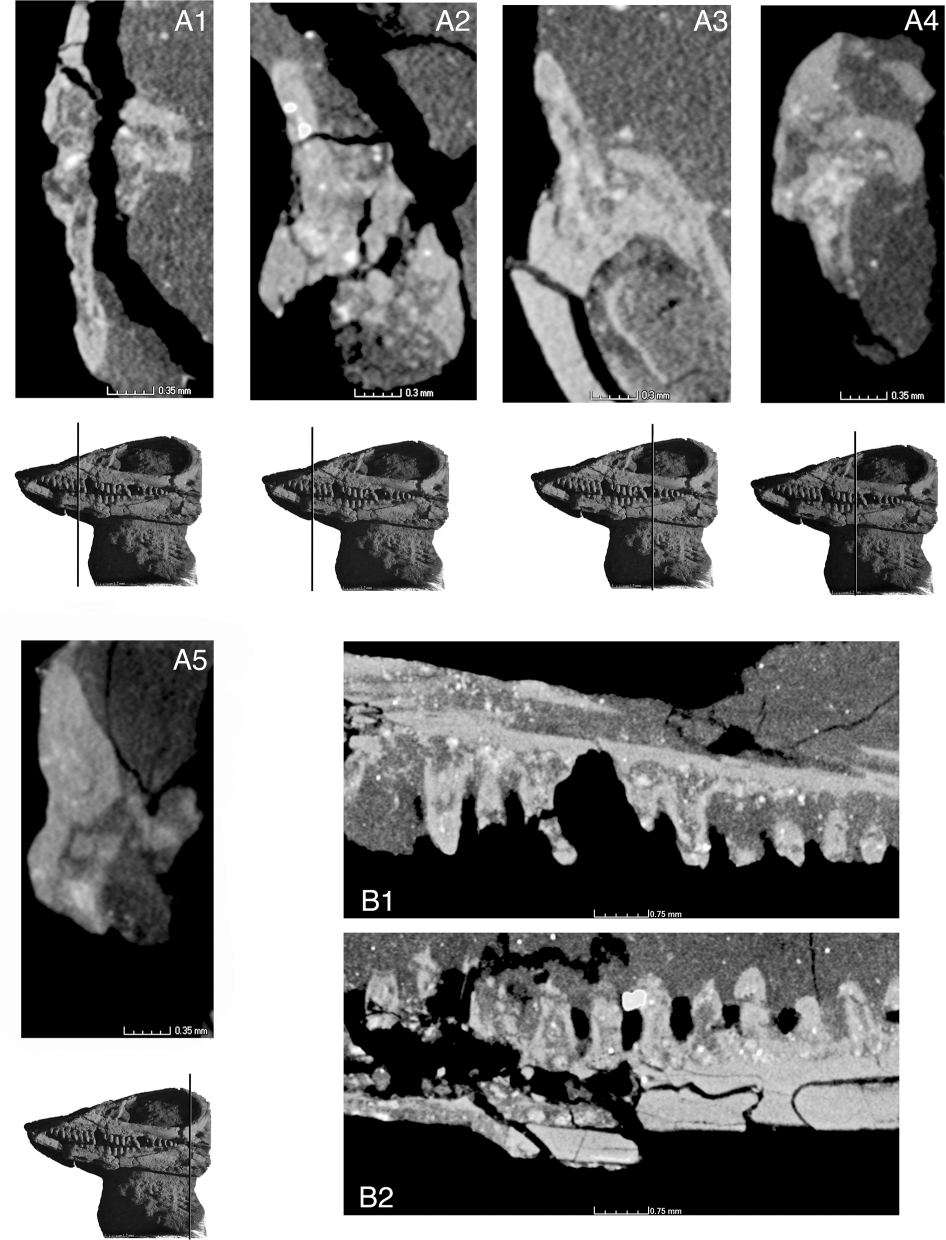
Tooth implantation in *Elachistosuchus huenei* MB.R. 4520 (holotype). Cross-sections of maxilla (A1 and A4; scale bars equal 0.35 mm) and dentary (A2, A3, and A5; scale bars equal 0.3 mm) teeth with respective planes of section indicated on the skull in left lateral view. B1-2, sagittal sections of B1, maxillary and B2, dentary tooth rows. Scale bars equal 0.75 mm.

The type of tooth implantation (Fig 6) varies slightly depending on the tooth-bearing bone and the relative tooth position. No caniniform region can be identified, and, more posteriorly, the maxillary tooth crowns become slightly more robust with broader bases. The dentary and maxillary teeth are cylindrical, and the more anterior ones possess a slight constriction at about two-thirds of their height. They have rather well developed roots and sit in shallow sockets, with neither the labial nor lingual wall being more developed up to a point at about the mid-portion of the tooth rows (Fig 6A-1 and 6A-2). At this point, the dentary sockets become shallower and the labial wall is slightly taller than the lingual one (Fig 6A-3). The maxillary socket walls, on the other hand, remain mostly unchanged in relation to one another (Fig 6A-4), but the sockets become increasingly shallow, and the two posterior teeth sit only superficially on the jaw bone (Fig 6A-5). The individual sockets have low or no bony partitions between them (Fig 6B).

### Vertebral Column

Janensch [5] identified the preserved humerus as the right one and based the orientation of the vertebrae on this interpretation. However, μCT scanning of this block revealed the presence of several previously unknown elements concealed within the matrix, the most important of which is a complete interclavicle. The position in which it is preserved indicates that the orientation of the articulated vertebrae was actually described backwards, and thus a redescription is provided here.

Janensch [5] identified six articulated *vertebrae* (Figs 2E and 6A–6H) plus one zygapophysis in the block containing the humerus. Scanning images show that part of its corresponding centrum is still buried in the matrix, as are two other elements anterior to the entire series (Fig 2J). The first and second vertebrae are represented only by their centra concealed in the matrix, the first one being poorly preserved with its anterior and posterior ends separated. The following three vertebrae are mainly represented by neural arches whereas their centra are poorly preserved. The sixth and seventh vertebrae possess both centrum and neural arch and are the best-preserved elements of the series. The last vertebra is the least complete: only the posterior end of its centrum and the prezygapophysis (rather than the postzygapophysis) are present almost in articulation with the preceding vertebra. Based on their preserved position in relation to the other elements of the block, these vertebrae can be identified as posterior cervicals and anterior dorsals. Janensch [5] identified them simply as being presacrals; he referred specifically to the vertebrae in block III as trunk vertebrae but did not identify their position more specifically. Janensch also referred to the ribs on this block as cervical ribs, but does not mention the associated vertebrae.

**Fig 7:**
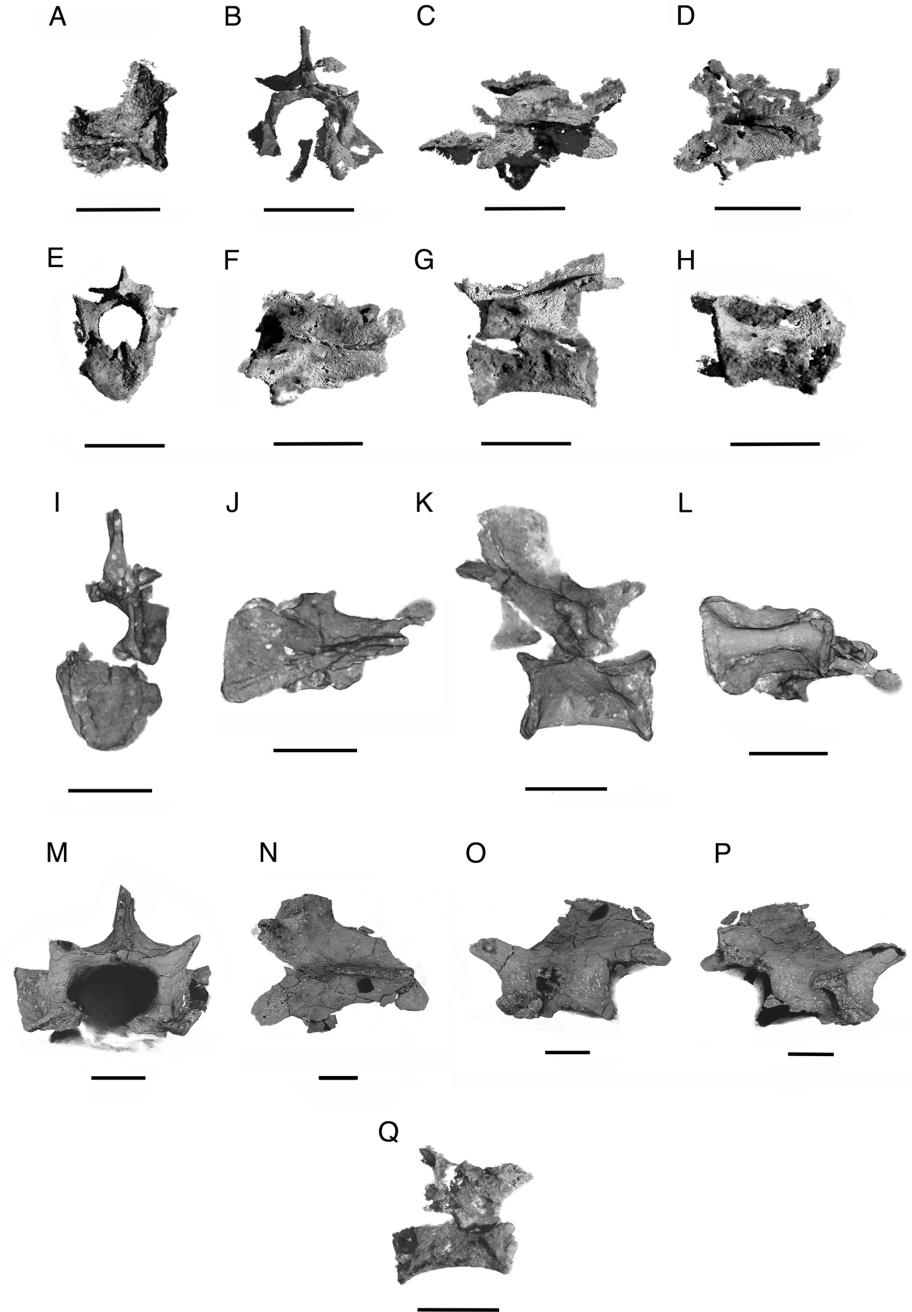
*Elachistosuchus huenei* MB.R. 4520 (holotype). Vertebrae. A, second vertebra of the series of block II (consisting of the centrum only) in dorsal view. B and C, third vertebra of block II in anterior and dorsal views. D, fourth vertebra of block II in dorsal view. E–G, sixth vertebra of block II in anterior, dorsal and left lateral views. H, seventh vertebra of block II in ventral view. I–L, the most complete vertebra of block III in posterior, dorsal, left lateral and ventral views. M–P, one of the prepared vertebrae of block III (consisting of the neural arch only) in anterior, dorsal left lateral and right lateral views. Q, sixth trunk vertebra in right lateral view. Scale bars equal 3 mm (A–H, Q) and 1.5 mm (M–P).

The vertebrae resemble those of basal diapsids such as *Araeoscelis* [39] but are not as elongate. The platycoelous centra are not keeled ventrally and are slightly dorsoventrally compressed at mid-length, so that in transverse section the centrum has a more oval outline. As in *Araeoscelis* and *Jesairosaurus* [40], the posterior articular surfaces of the fifth and sixth centra are slightly inclined anteriorly, but the posterior surface of the seventh is vertical. There is no ventral bevelling for contact with intercentra nor is there a significant change in size along the series, with centrum length measuring about 4.4 mm. On the right side of the centrum of the sixth vertebra, an articular facet for a rib is located on the anterior part of the centrum (Fig 7Q). None of the preserved neural arches has a prominent transverse process, but here the facet has dorsal and ventral parts that are connected to each other at a right angle, similar to *Youngina*. Although there appear to be a distinct parapophysis and diapophysis, the latter is not situated on the neural arch. Taking into account the presence of distinct but closely spaced capitulum and tuberculum (see below), a transition to a single synapophysis for the articulation with the rib head was present in *Elachistosuchus*, as in *Clevosaurus* [25].

The centra are not tightly fused to their neural arches; this is clearly evident on the right side of the sixth vertebra. In transverse section, the pedicles connecting the neural arch to the centrum extend down in a rounded rather than straight manner, enclosing between them a wide and low neural canal, which is very similar to that in *Gephyrosaurus* and *Sophineta* [23]. In dorsal view, the prezygapophyses diverge slightly from the midline, at angles less than 45°, whereas the postzygapophyses diverge more sharply, resembling the condition on the dorsal vertebrae of *Clevosaurus*. The pre- and postzygapophyses are subequal in length. The prezygapophyses are set distinctly lower than the postzygapophyses, forming a sinuous contour in lateral view. This peculiar mode of articulation between vertebrae has also been reported in *Petrolacosaurus* [41] and *Clevosaurus* [25]. The zygapophyses do not extend much beyond the centrum either laterally or anteroposteriorly. None of the neural spines is completely preserved but a thin median ridge can be seen extending along the dorsal surface of the neural arches. The absence of complete fusion of the neural arches to the centra and the proportionately long interclavicle (the length of which is 3.6 times that of a dorsal vertebra; [42]) indicate the juvenile nature of the holotype of *Elachistosuchus huenei* as suggested by Janensch [5].

**Fig 8:**
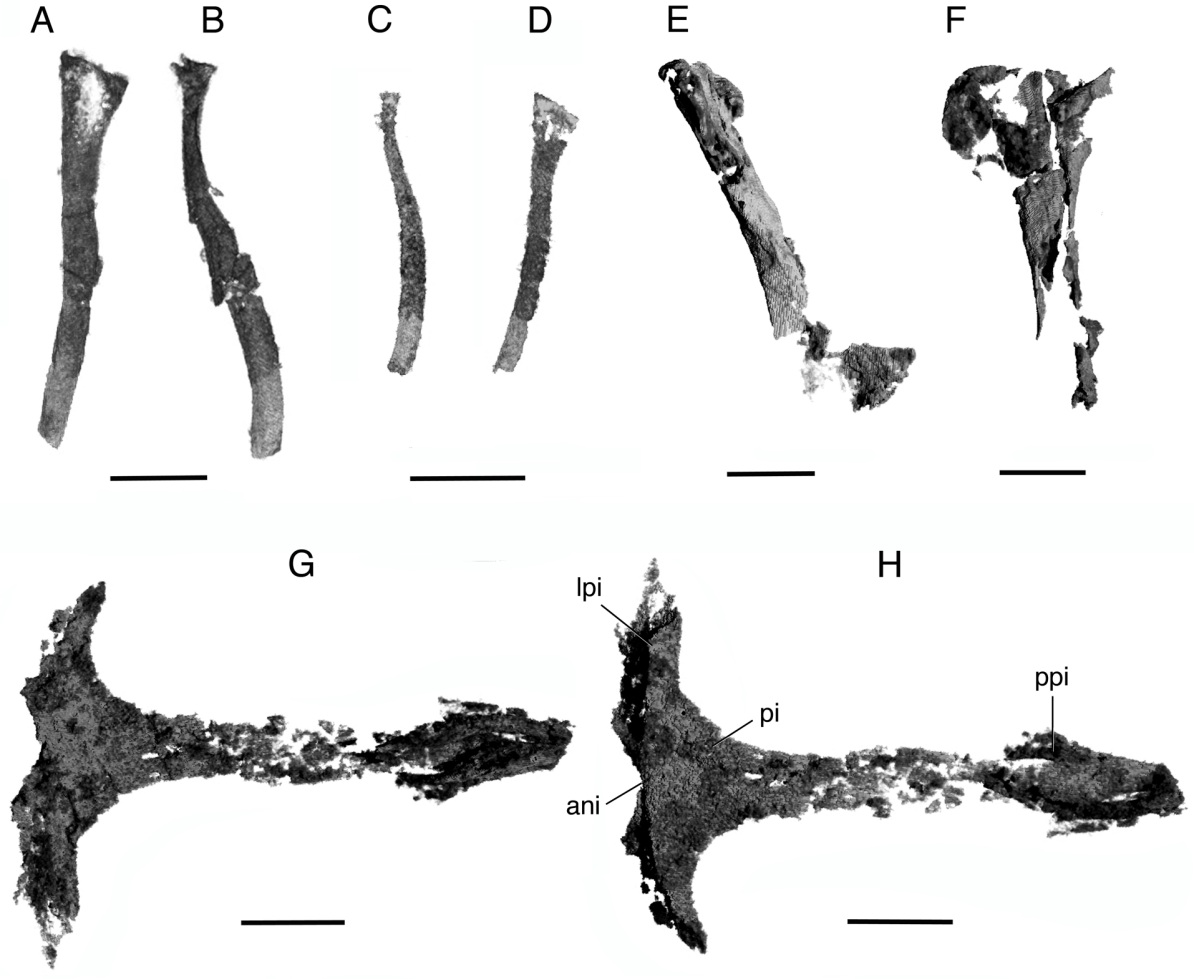
*Elachistosuchus huenei* MB.R. 4520 (holotype). A, B, rib of block III in A, dorsal and B, lateral views. C, D, rib of block IV in C, lateral and D, dorsal views. E, F, left humerus in E, lateral and F, posterior views. G, H, interclavicle in G, dorsal and H, ventral views. Scale bars equal 3 mm (A, B, E–H) and 5 mm (C, D), respectively. Abbreviations: ani, anterior notch of interclavicle; lpi, lateral process of interclavicle; pi, plate of interclavicle; ppi, posterior process of interclavicle.

Janensch [5] reported seven vertebrae preserved in block III, three of which were identified as caudals. Of the remaining presacral ones, two have been extracted from the block: one without the centrum and the other so well preserved that it was used as the basis for his description. However, only four vertebrae were actually found in the block, all partially exposed on the surface. Two centra without associated neural arches were identified as probably belonging to the tail, but none of them belongs to the caudal series. Furthermore, they are not even remotely preserved close to each other, let alone next to one another as reported [5:235]. Of the prepared vertebrae, the one represented only by the neural arch is readily identifiable, whereas the identification of the other is more dubious. A small piece of bone was found close to block III, but closer examination and scanning images show that it does not correspond to the vertebra illustrated by Janensch [5:232]. It is a poorly preserved element with a partial neural arch and centrum. There is a label associated with the specimen made, or following notes, by Michael J. Benton (dated 2.5.83) referring to the vertebrae illustrated by Janensch [5] (the isolated complete dorsal in Figs 6–8 and one “caudal” in Fig 12, both in [5]), but, instead of the corresponding vertebrae, there are only two unprepared blocks of matrix. Apparently, one “caudal” was later removed from block III, but both this and the complete dorsal vertebra were subsequently lost. The position of the newly identified, partially preserved vertebra in the vertebral column is uncertain.

Of the four vertebrae preserved in the block, two are represented only by their centra (one of which is complete), one is a fragmentary partial centrum with associated neural arch, and the fourth is a fairly complete vertebra lacking most of the left side of the neural arch (Fig 7I–7L). The centrum of the most complete vertebra is 5.36 mm long and has a minimum height of 2.35 mm and the complete one without part of the neural arch is 4.41 mm long and has a minimum height of 2.51 mm. Considering that they were preserved in association with several ribs, and based on their structure (see below), they probably represent mid-dorsal vertebrae. The centra closely resemble those described previously, but the associated neural arches differ significantly. The difference in height between the pre- and postzygapophyses is much more marked. The facets for rib articulation are also more clearly separated from each other. The transverse process is somewhat prominent, extends perpendicular, and is inclined anteroventrally, whereas the parapophysis is posterodorsally directed and situated far anteriorly on the centrum. The articular facets were certainly connected to one another and are aligned on the lateral side of the vertebra. One fairly well preserved neural spine is tall, being higher than the centrum (3.3 mm vs. 2.3 mm). Its anterior margin is slightly recurved, forming a contour similar to the one between the zygapophyses. The neural canal is less flattened.

Of the prepared vertebra, only the isolated neural arch can be identified among the labelled material (Fig 7M–7P). The transverse processes are more prominent and extend more parallel to the centrum than on the previous one. There is an anteroventrally extending connection to the more ventrally situated (now missing) parapophysis. The differences between the positions of the zygapophyses are not as pronounced as on the neural arches described above. Furthermore, the neural spine rises less steeply and is lower. The neural canal is more similar to that of the anterior dorsal vertebrae, but it is broader.

The vertebral remains of block IV consist of an incomplete centrum and a partial neural arch with an associated centrum. The preservation of the elements is not particularly good and they add little to what has already been described. The neural arch is similar to the one of the previous block, but the neural canal is markedly more rounded in end view and taller. The preserved transverse processes are short and extend horizontally from the neural arch laterally.

Based on his incorrect orientation of block II, Janensch [5] attributed the *ribs* to the cervical region but they more likely represent anterior dorsal ribs. He identified three preserved ribs on the right side, but part of a fourth rib is concealed in the matrix. The poor preservation does not allow for a detailed description, and there is little to add to the original account. The ribs are thin, very long (about 5 times the length of a centrum), slightly dorsoventrally flattened, and gently curved. Only one of the ribs has a well-preserved proximal end. It is dichocephalous, with the capitulum and tuberculum placed closely together, with virtually no separation and similar to the structure of the seventh rib of *Prolacerta* [17:109]. The proximal portion of the rib is not twisted, and a shallow groove extends along the anterior border of the shaft. No uncinate or accessory processes are present.

Between 15 and 17 ribs were counted inside block III, but as several are represented only by small, uninformative pieces, this number may represent an overestimate. In transverse section, the ribs are flattened to varying degrees and much more robust than the ones in block II. Only one of them preserves a flattened, triangular proximal end with two distinct facets for articulation with the corresponding vertebra (Fig 8A and 8B). A groove extends along one of the borders of the shaft. A second, fairly well preserved rib shows a different structure, with less separation between the articular facets, based on the form of the proximal part of the shaft and the preserved part of the head.

The six ribs of block IV are delicate and poorly preserved. They resemble one of the ribs in block III, with an expanded triangular head and a longitudinal groove along the shaft (Fig 8B and 8C).

Block V contains only fragments of ribs and/or gastralia. Janensch [5] identified a metatarsal among these fragments but it cannot be located now. Block VI apparently lost a small piece. On the main block there are only a few fragments of bone, whereas the broken-off bit contains more bone.

### Pectoral Girdle and Appendicular Skeleton

The μCT scans revealed for the first time the *interclavicle* (Figs 2K and 7G and 7H), which is completely concealed in the matrix. The bone is T-shaped with a broad anterior plate between the anterior transverse bar and the posterior process, similar to the rhomboid interclavicles of basal synapsids [19, 21], of *Youngina* [17], and *Tasmaniosaurus* [43]. Its lateral processes are long and gently curved posteriorly, and their anterior margins are slightly concave. The anteroventral margins of the lateral processes bear deeply recessed surfaces for reception of the clavicles. Between the clavicular facets, the anterior margin of the interclavicle has a distinct, broad notch, as in *Prolacerta* [17], *Mesosuchus*, and other archosauromorphs [44]. The lateral processes have a combined width of 10.5 mm. The interclavicle bears a long and rather broad posterior process, which measures 12.82 mm out of a total length of 16.35 mm of the interclavicle. The posterior process does not taper significantly posteriorly, terminating in a rather blunt tip, as in *Prolacerta* and *Macrocnemus* [45]. Halfway to its posterior end, the stem becomes wider so that the overall outline of the process becomes spatulate, resembling the interclavicle of *Mesosuchus* [44]. The length of the lateral process equals about one third of that of the posterior process. In lateral view, the anterior and posterior portions of the interclavicle curve gently dorsally, so that the anterior, notched border and the posterior end of the posterior process lie on approximately the same level, similar to the condition in *Clevosaurus* [25].

Both *clavicles* (Fig 2K) are preserved in the matrix. The right clavicle is only partially preserved at its medial end and is displaced anteriorly. The left bone is complete, and, although no longer in articulation with the interclavicle, lies close to its original position. The clavicle is a rather thin element, with a narrow dorsal process and an expanded medial portion that points posteriorly. The ends are connected by a sigmoidal shaft that bears a depression for the contact with the anterior edge of the scapula along the posterior margin of its ventral surface. Although still fairly different, this structure is more similar to that of the clavicles of *Araeoscelis*, *Petrolacosaurus*, or *Prolacerta*. In contrast, the clavicles of *Gephyrosaurus* are dorsally concave. The clavicle is gently concave ventrally.

Associated with the girdle elements, there is an incomplete long bone identified here as a *scapula* based on its association with the pectoral girdle (Fig 2K). The bone is straight and strongly dorsoventrally compressed. One of its ends is relatively broad and probably represents the distal portion. The bone tapers slightly towards the other, presumably proximal end. A pronounced constriction is present close to this other end, from which it expands slightly again. In ventral view, this other end apparently bears a concavity, possibly the glenoid facet of the scapula. Other bones of the pectoral girdle were found and segmented (Fig 2K), but, due to their incomplete preservation, it is not possible to identify them with confidence.

As noted above, Janensch [5] interpreted the *humerus* as the right element because, he argued, its flat distal portion would expand toward the medial side. He offered no additional explanation for this interpretation. The discovery of bones of the pectoral girdle, however, suggests that the humerus is, in fact, the left one. As most of the humerus (Fig 8E and 8F) is exposed and much of it is not well preserved, little can be added to the description by Janensch [5]. The strong twist (about 90°) between distal and proximal ends of the humerus is also present in neodiapsids such as *Askeptosaurus* [37] and *Youngina* [17], basal lepidosauromorphs such as *Gephyrosaurus* [46] and *Clevosaurus* [25], and basal archosauromorphs as *Prolacerta* [17]. The length of the humerus is 14.22 mm, and thus the interclavicle is only 12% longer than the humerus. The shaft of the humerus is 1.7 mm wide at mid-length.

A small piece of matrix lying close to the block with the humerus was found in the box with the remainder of the material. They were likely not originally associated, but association of the small piece with any of the other blocks is also uncertain. It contains poorly preserved rib or gastralia fragments, which add no useful information.

## Phylogenetic Relationships

**Fig 9:**
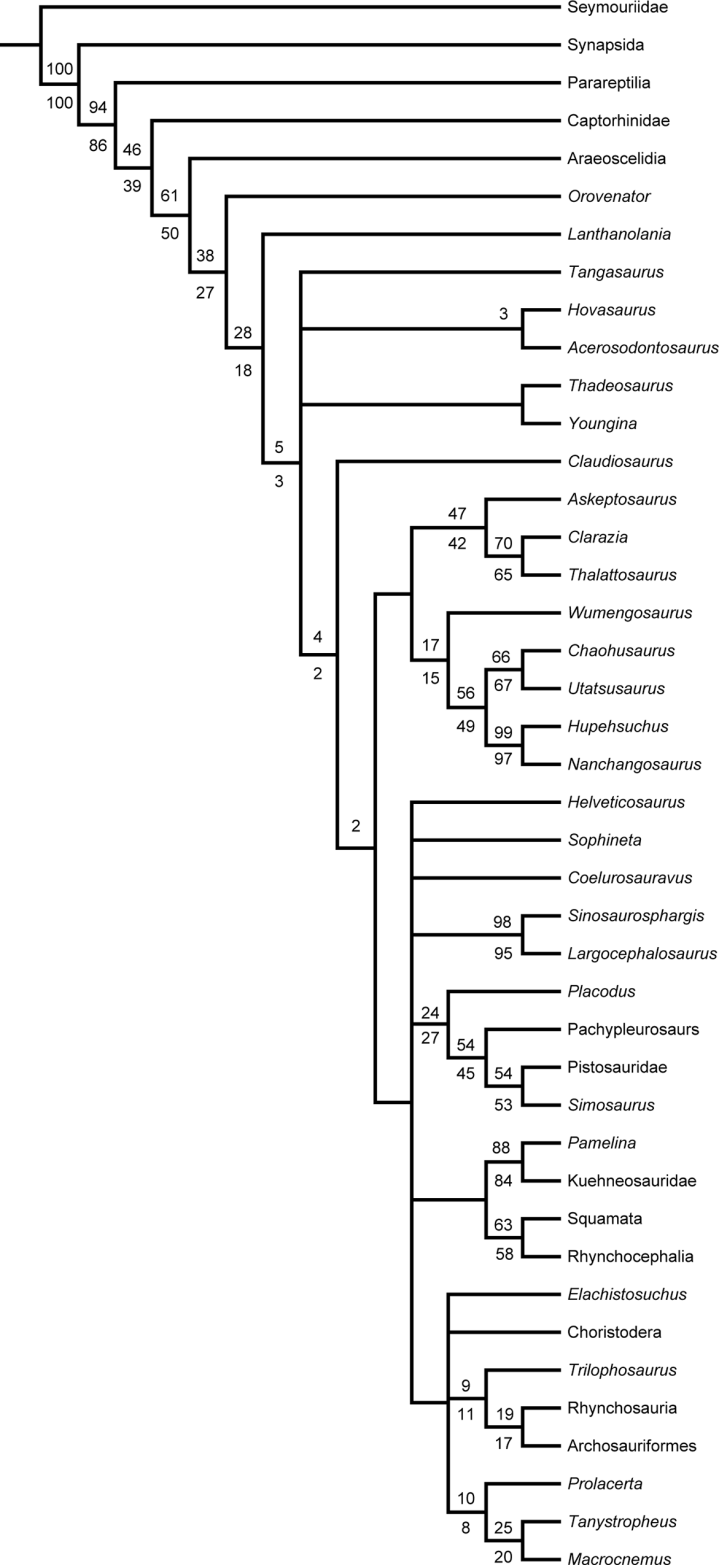
Strict consensus tree of 12 most parsimonious trees, each with a length of 810 steps, from the phylogenetic analysis using the character-taxon matrix of Chen *et al*. [12] with *Elachistosuchus huenei* included. Numbers above branches indicate bootstrap values. Numbers below branches indicate jackknife values > 50. Bremer support values were lower than 0 for all branches. CI = 0.314 and RI = 0.581.

The phylogenetic analysis with inclusion of *Elachistosuchus* in the character-taxon matrix of Chen *et al*. [12] resulted in 12 most parsimonious trees with a length of 810 steps each and with a consistency index of 0.314 and a retention index of 0.581. Overall, the topology has a lower resolution around the node of Sauria than the one obtained in Chen *et al*. [12]. In comparison to the topology published by Chen *et al*. [12], in the strict consensus tree (Fig 9), the base of Sauria is collapsed together with *Sophineta* + *Coelurosauravus*, although some monophyletic clades are recovered, such as Saurosphargidae, Sauropterygia, Lepidosauromorpha, and Archosauromorpha. The latter is also collapsed at its base, where *Elachistosuchus* clusters with Choristodera, “Prolacertiformes”, and *Trilophosaurus* + (Rhynchosauria + Archosauriformes). *Elachistosuchus* has 11 autapomorphies: clavicles broad medially (character 52, 1 → 0), orientation of basipterygoid processes lateral (character 96, 0 → 1), most trunk ribs dichocephalous (character 104, 1 → 0), maxillary tooth row extending backwards beyond posterior margin of orbit (character 118, 0 → 1), scapula slender, high, and narrow (character 119, 0 → 1), maxilla orbital exposure present (character 128, 0 → 1), sphenethmoid present (character 138, 1 → 0), frontal length no greater than twice the width (character 155, 0 → 1), paroccipital processes extending laterally forming 90° with parasagittal plane (character 158, 1 → 0), prearticular extending anterior to coronoid eminence (character 168, 1 → 0), and frontal butterfly-shaped with anterolateral and posterolateral processes (character 202, 0 → 1).

**Fig 10:**
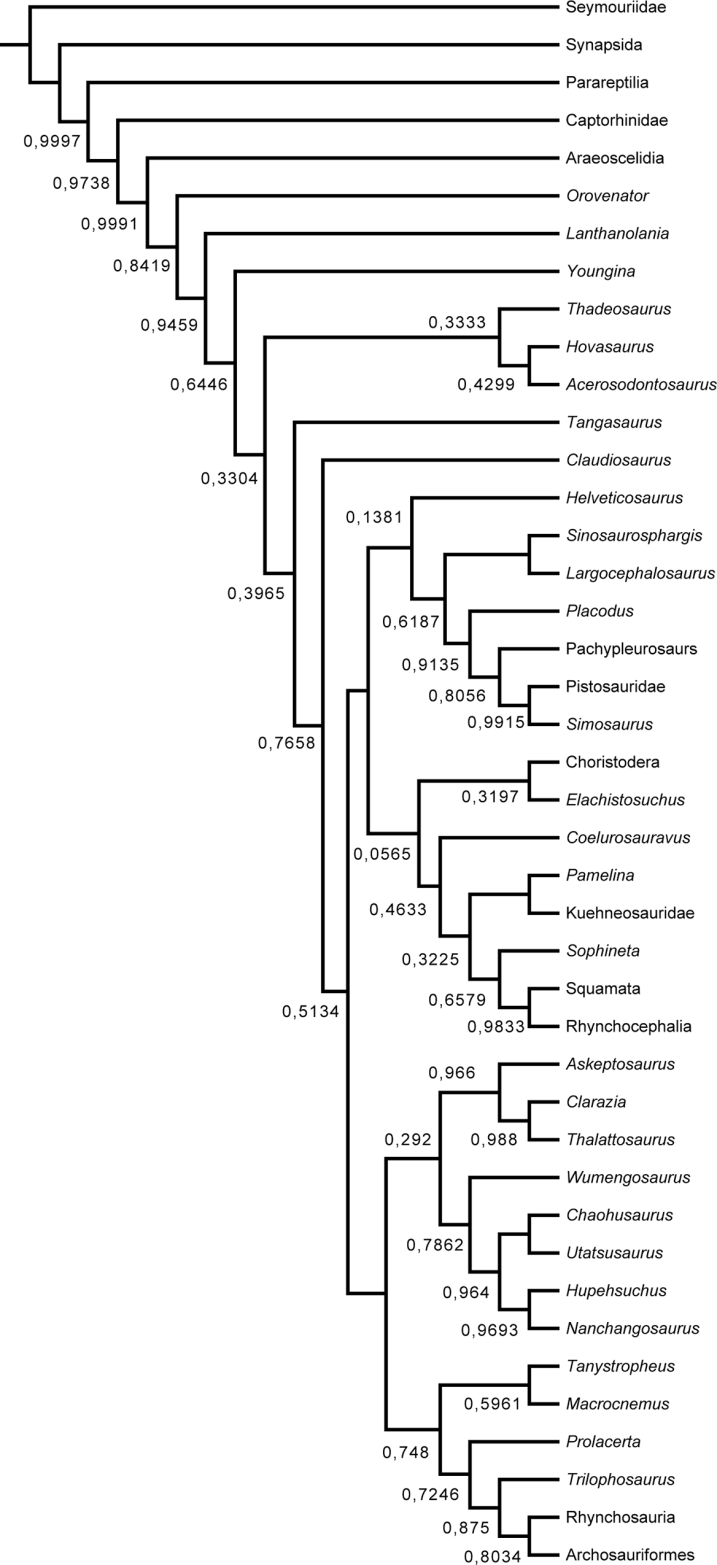
"Allcompat" consensus tree from the Bayesian analysis using the character-taxon matrix of Chen *et al*. [12] with *Elachistosuchus huenei* included. Numbers indicate the posterior probabilities of branches. Nodes without number indicate a posterior probability of 1.

The Bayesian analysis (Fig 10) found a substantially different topology from the one retrieved by maximum parsimony, where *Elachistosuchus* is retrieved as the sister-taxon to Choristodera and both taxa have lepidosauromorph affinities. The posterior probabilities supporting this relationship, however, are very low (0.0565). Probabilities of all clades at the saurian node except for Archosauromorpha, Thalattosauria, *Wumengosaurus* + (Hupehsuchia + Ichthyopterygia), Saurosphargidae + Sauropterygia, and *Sophineta* + Lepidosauria are also less than 0.5. In this scenario, a clade including Thalattosauria, *Wumengosaurus*, Hupehsuchia, and Ichthyopterygia has archosauromorph affinities, whereas *Helveticosaurus* and the Sauropterygia + Saurosphargidae clade are placed closer to Lepidosauromorpha. We also performed an additional Bayesian analysis of the original dataset without *Elachistosuchus* (Supporting Information–S1 Fig), which we briefly describe here, focusing only on the relevant changes. Without inclusion of *Elachistosuchus*, Choristodera is related to Archosauromorpha, as is Thalattosauria. Saurosphargidae + Sauropterygia and *Wumengosaurus* + (Hupehsuchia + Ichthyopterygia) form a big “marine clade” with archosauromoph affinities as well. The posterior probabilities at the base of Sauria are again very low. Basically all the abovementioned groups are retrieved with certainty, whereas the lepidosauromorph affinities of *Coelurosauravus* also receive stronger support. Thus, the addition of *Elachistosuchus* moves Choristodera to a less-accepted position but splits the likely artificial large clade of marine reptiles [12] while keeping the clade Thalattosauria + (Hupehsuchia + Ichthyopterygia) intact. In comparison with the original analysis of Chen *et al*. [12], both Bayesian approaches retrieve this latter clade within Sauria–a similar result to that found by Chen *et al*. [12] when characters considered aquatic adaptations were not scored as ambiguous.

**Fig 11:**
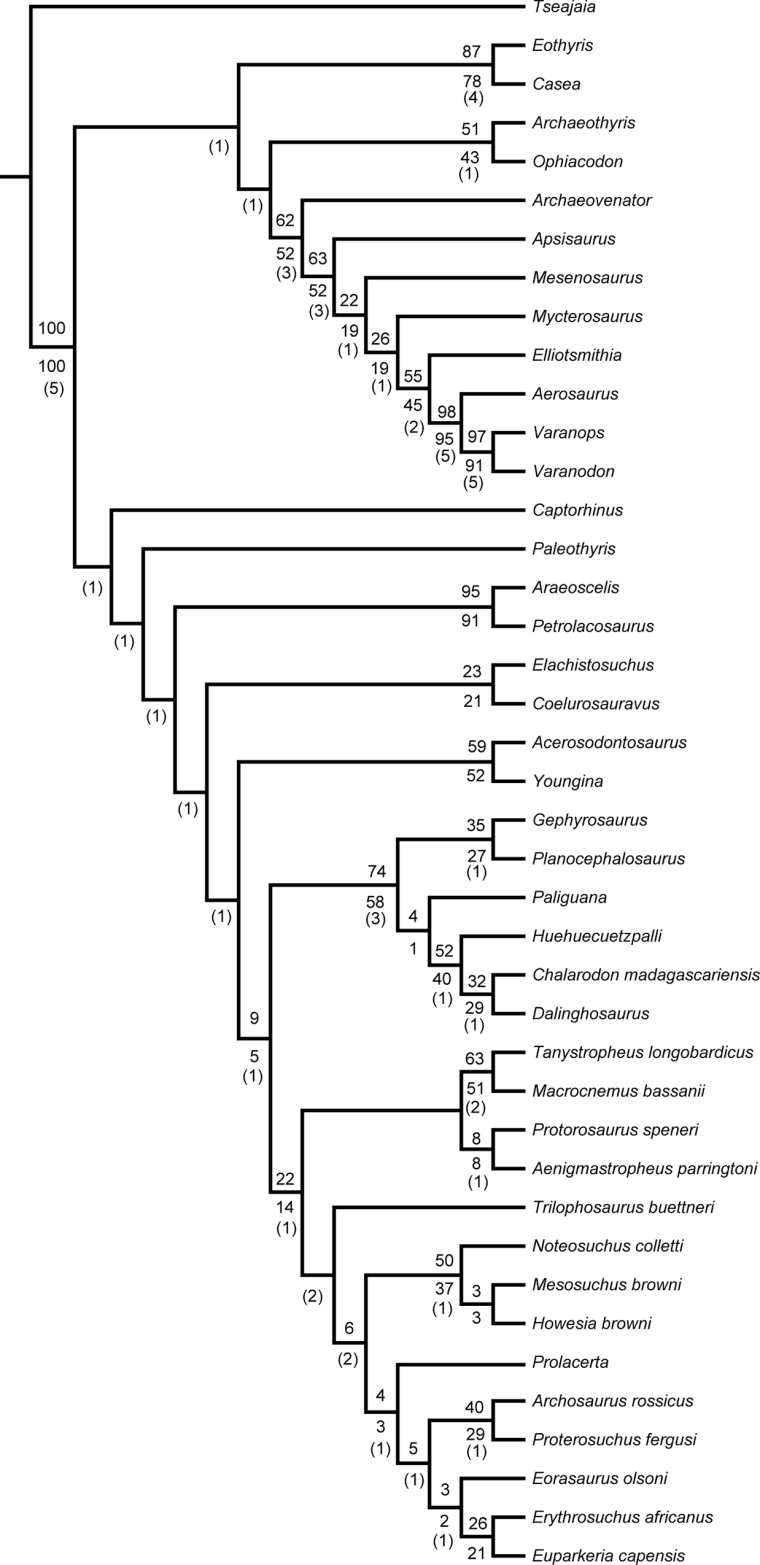
Single most parsimonious tree with a length of 880 steps from the phylogenetic analysis using the character-taxon matrix of Ezcurra *et al*. [13] with *Elachistosuchus huenei* included. Numbers above branches indicate bootstrap values. Numbers below branches indicate jackknife values > 50. Bremer support values underlined. CI = 0.330 and RI = 0.641.

It could be argued that the matrix of Chen *et al*. [12] is biased towards taxa with features viewed as adaptations for an aquatic mode of life, and thus we decided to include *Elachistosuchus* in the only other recently published character-taxon matrix for basal diapsids and early saurians [13], which focuses on terrestrial taxa. The analysis resulted in a single most parsimonious tree with a length of of 880 steps, a consistency index of 0.330, and a retention index of 0.641 (Fig 11). The topology is almost the same as the one published except it is now fully resolved. In this analysis, *Elachistosuchus* is the sister-taxon of *Coelurosauravus*, and this clade is supported by five unambiguous synapomorphies: postorbital part of the skull longer (1.03 mm) than antorbital (0.97 mm) part (character 6), maxilla orbital exposure present (character 123), postaxial cervical intercentra absent (character 168), dorsal intercentra absent (character 175), and length of the anterior dorsal centra more than two times the height of the centrum (character 176). *Elachistosuchus* has 11 autapomorphies: distal curvature of marginal teeth absent (character 2), supratemporal absent (character 51), angular lateral exposure narrow (character 61), notochordal canal absent in adults (character 67), transverse process of dorsal vertebrae moderately long (character 74), anterior margin of the scapula convex along its entire length (character 85), maxilla posterior extension reaching level or posterior to posterior orbital border (character 122), frontal suture with nasal oblique, with nasal extending considerably between frontals in a non-interdigitating suture (character 128), skull roof with distinct posterior emargination in late ontogeny (character 134), presence of a distinct dorsal process behind the alveolar margin on the lower jaw formed by a dorsally well-developed coronoid and sometimes the posterodorsal ramus of the dentary (character 158), and cervical ribs slender and tapering at low angle to vertebrae (character 173).

**Fig 12:**
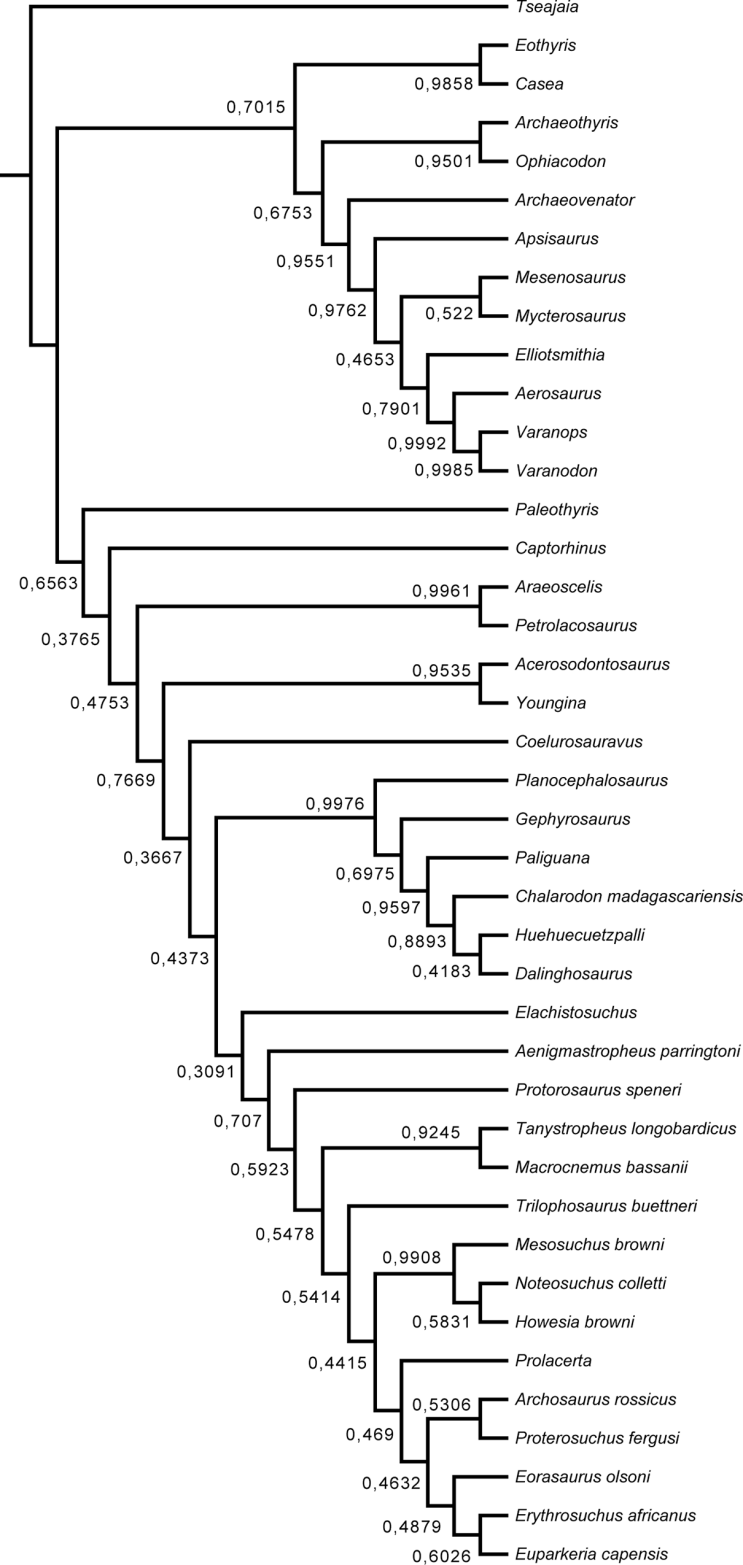
"Allcompat" consensus tree from the Bayesian analysis using the character-taxon matrix of Ezcurra *et al*. [13] with *Elachistosuchus huenei* included. Numbers indicate the posterior probabilities of branches. Nodes without number indicate a posterior probability of 1.

The result of the Bayesian analysis shows a significant change in the relationship of *Elachistosuchus* (Fig 12). *Coelurosauravus* is still a non-saurian diapsid but now is placed as the sister-taxon of Sauria, and *Elachistosuchus* is retrieved as the basalmost archosauromorph. Again, the posterior probabilities around the saurian node are very low. The phylogenetic relationships within the clades are also slightly different from the original results: *Planocephalosaurus* basal to *Gephyrosaurus*, *Chalarodon* in Squamata instead of *Huehuecuetzpalli*, *Aenigmastropheus* and *Protorosaurus* as successive taxa at the base of Archosauromorpha, and *Mesosuchus* in Rhynchosauria instead of *Noteosuchus*. These changes are, however, not due to the addition of *Elachistosuchus* (Supporting Information–S2 Fig). The inclusion of *Elachistosuchus* in the Bayesian analysis also results in the recovery of *Coelurosauravus* as a diapsid more closely related to Sauria.

Because the Bayesian approach used unordered characters, an additional parsimony analysis of the matrix of Ezcurra *et al*. [13] was performed with all characters unordered as well, with and without the exclusion of *Elachistosuchus* (Supporting Information–S3 and S4 Figs). In comparison to the original result published by Ezcurra *et al*. [13], the unordered matrix collapses the amniote node, where the following clades are retrieved in a polytomy: *Petrolacosaurus* + *Araeoscelis*, *Paleothyris*, *Captorhinus*, and the remaining taxa of Diapsida. Unordering the characters also collapses *Coelurosauravus* and *Youngina* + *Acerosodontosaurus*. It also collapses proterosuchid and archosauriform taxa. Addition of *Elachistosuchus* retrieves it as the sister-taxon of *Coelurosauravus*, both together forming the sister-group of Sauria.

The first point to be noted is the dramatically different positions of *Elachistosuchus* in the analyses. The inclusion of *Elachistosuchus* in the matrix of Chen *et al*. [12] yields a close relationship of this taxon either to Archosauromorpha or to Lepidosauromorpha, but the inclusion of *Elachistosuchus* in the matrix of Ezcurra *et al*. [13] recovers this taxon as a basal diapsid forming a monophyletic group with *Coelurosauravus* in one analysis and as a basal archosauromorph in another. The two data sets were used to assess the phylogenetic relationships of *Elachistosuchus* because they represent the most recent and complete reviews for this part of the diapsid Tree of Life. Chen *et al*. [12] used a matrix that focuses on neodiapsid clades more basal than the saurian node, whereas that of Ezcurra *et al*. [13] concentrated on basal archosauromorph taxa. In the former *Elachistosuchus* is retrieved as a basal saurian, but in the latter its position is contradictory. Recovering *Elachistosuchus* as the sister-group of *Coelurosauravus* outside Sauria in the parsimony analysis of the matrix of Chen *et al*. [12] takes 4 or 7 additional steps, depending on the topology. However, in our analysis, *Coelurosauravus* is found in the polytomy that includes the origin of Sauria, a considerably more derived position in comparison to Ezcurra *et al*. [13]. Moving both clades to a position more basal than *Youngina* results in a tree 18, 21, or 22 steps longer. Having *Elachistosuchus* as the basalmost archosauromorph in the matrix of Ezcurra *et al*. [13] takes 5 additional steps, but moving it and *Coelurosauravus* to be the sister-group of Sauria results in a tree only 2 steps longer. Recovering *Coelurosauravus* + *Elachistosuchus* as the basalmost clade of Lepidosauromorpha (as found in some of the most parsimonious trees of the analysis of the matrix of Chen et al. [12], as well as in the Bayesian analysis) takes 12 additional steps.

Although the Bayesian results differ from the parsimony ones in either case, *Elachistosuchus* seems to have a significant impact on the phylogenetic position of the aquatic clades as its inclusion yields results that are in better accordance with the most recent proposals for the relationships of these groups [12]. Whereas the Bayesian results of the analyses of Chen *et al*. [12] and Ezcurra *et al*. [13] do not agree on the specific saurian affinities of *Elachistosuchus*, they both recover it as closely related to the saurian node. It is therefore safe to conclude that *Elachistosuchus* is a taxon with affinities close to the origin of Sauria, and that the instability concerning its position highlights our poor understanding of this node. In any case, despite the uncertainty about the position of *Elachistosuchus* among diapsids, our results reject previous hypotheses about its classification. Although a possible relationship with basal archosauriforms cannot be ruled out, previous interpretations of *Elachistosuchus* as a pseudosuchian archosaur [5] or as a rhynchocephalian lepidosaur [6] are clearly not supported by any of the analyses.

The four different and conflicting topologies indicate that the datasets fail to properly distinguish between plesiomorphic and apomorphic characters for the saurian node. Thus, it is worth highlighting the importance of morphological reassessments of poorly known taxa, as done in the present study, for understanding the evolution of morphologically disparate clades such as the various groups of Mesozoic marine reptiles and *Coelurosauravus*. There is an urgent need for morphologically and temporally transitional fossils that could help elucidate not only the phylogenetic relationships of these taxa but also their early evolutionary history–and the current lack of such forms is partially responsible for the unstable position of many taxa in different phylogenetic hypotheses.

The second point to be mentioned is the overall differences of the topologies produced by the analyses of both data sets. Given the reduced number of overlapping taxa between the datasets, the discussion will focus on Archosauromorpha and *Coelurosauravus* only. The parsimony-generated tree based on Chen *et al*. [12] recovers *Coelurosauravus* as a saurian likely close to Lepidosauromorpha. By contrast, the topology of Ezcurra *et al*. [13] recovers *Coelurosauravus* as one of the basalmost diapsids. Results based on Chen *et al*. [12] show a monophyletic “Prolacertiformes” basal to a clade with *Trilophosaurus* and Rhynchosauria + Archosauriformes. However, in the Bayesian approach, *Tanystropheus* + *Macrocnemus* are recovered basal to *Prolacerta*. Similarly, in the trees based on Ezcurra *et al*. [13], Prolacertiformes is not monophyletic, with *Prolacerta* being more derived than the *Tanystropheus* + *Macrocnemus* clade. Closer to Archosauriformes, however, the relationships change quite dramatically. The results based on Chen *et al*. [12] show a closer affinity between Archosauriformes and Rhynchosauria, while the ones based on Ezcurra *et al*. [13] find a closer relationship between Archosauriformes and *Prolacerta*. The relative position of *Trilophosaurus* also varies between the two datasets, being closer to Archosauriformes in the results based on Chen *et a*l. [12] than in the ones based on Ezcurra *et al*. [13].

It could be argued that the differences in focus between the two matrices (aquatic vs. terrestrial taxa) are the source of conflicting topologies. Features related to aquatic habits are generally interpreted as secondary adaptations. Therefore, various aquatic diapsid groups have frequently been excluded from phylogenetic analyses of Diapsida. There are, however, reasons to believe that aquatic or semiaquatic habits were more widespread among eureptiles than traditionally assumed [47] and that the exclusion of aquatic or semi-aquatic taxa biases the analyses. This could partially explain the differences in the two topologies obtained.

The recovery of *Elachistosuchus* (Late Triassic) at the base of, or closely related to, Archosauromorpha does not help filling in the current temporal gap in the early evolutionary history of the group (prior to the Late Permian). Its recovery, however, as a sister-taxon to Choristodera (first known from the Middle Jurassic) in the Bayesian analysis of the matrix of Chen *et al*. [13] partially fills the temporal gap between Choristodera and other lepidosauromorphs, suggesting Choristodera might have originated much earlier than previously thought. The basalmost and oldest known choristodere is *Cteniogenys* from the Middle to Late Jurassic of Europe and the Late Jurassic of North America [48], although the poorly known *Pachystropheus* has been tentatively assigned to the group [49], which would extend the temporal range of Choristodera back to the Late Triassic (Rhaetian). However, this scenario still leaves a significant gap in the early evolutionary history of Lepidosauromorpha.

The addition of *Elachistosuchus* to the matrix of Ezcurra *et al*. [13] creates a considerable ghost lineage in the tree between it and *Coelurosauravus* (Late Permian). In this case, *Elachistosuchus* would be by far the youngest non-saurian basal diapsid ever reported. Likewise, its possible position more basal to *Aenigmastropheus* (Late Permian) also creates a significant ghost lineage at the base of Archosauromorpha. In any case, our results fail to provide a satisfactory answer regarding the apparent temporal gap in the evolutionary history of early saurians, which is particularly large on the lepidosauromorph side of the tree [13].

Besides indicating a gap in the known fossil record, the solution to fill in such gaps may well lie in the reassessment of such as *Elachistosuchus*, but especially more basal neodiapsids, both terrestrial and aquatic ones. The analyses of Chen *et al*. [12] and Ezcurra *et al*. [13] included taxa that were only recently described (*Sophineta*) or which are rarely considered (*Paliguana*), but still they ignored a handful of other important taxa such as *Drepanosaurus* or *Palaeagama*. Avoiding inclusion or pruning of poorly documented taxa is still practised based on the low percentage of identifiable characters [50–52], but a high number of plesiomorphies or conflicting character information may also result in unstable taxa [53–55]. It has been shown that the addition of incomplete taxa is actually important for phylogenetic analysis [56, 57] and that restricted taxon selection may be more harmful [51, 57, 58]. Outgroup selection can have a significant impact in ingroup rooting and topology, especially in cases of hard or near-hard polytomies [59, 60]. When added in insufficient numbers or represented by more derived taxa of related clades (long branches), outgroups usually fail to correctly polarize characters and cause long-branch attraction problems [61, 62].

It is unlikely that the origin and early diversification of Sauria will be resolved in the near future without morphological reassessment and incorporation of a greater number of the ‘problematic’ clades into phylogenetic analyses. Furthermore, the conflicting results could indicate a rapid speciation event early in the evolutionary history of the group. In cases where successive speciation events occurs simultaneously or in rapid succession, known as hard or near-hard polytomies [63], few synapomorphies accumulate in the internal branches. Even if a considerable amount of data is available, methods for phylogenetic reconstruction can fail to retrieve the correct relationships if the outgroup branches are longer than ingroup ones, making the analysis sensitive to outgroup selection and more prone to long-branch attraction [59, 60]. If we consider that key taxa such as turtles are likely to be related to basal saurians but that their precise phylogenetic positions still remain uncertain, the importance of getting a better resolution of saurian interrelationships is obvious. We do not yet understand the pattern in which plesiomorphic and apomorphic features evolved among early saurian taxa and much additional research on this issue is needed.

## Conclusion

We have redescribed the holotype and only known specimen of the long-forgotten enigmatic diapsid *Elachistosuchus huenei* from the Late Triassic of Germany using μCT scanning. With this technique we were able to retrieve new information such as details of the structure of the palate, basicranium, tooth implantation, and shoulder girdle, which were inaccessible up to now. It was also possible to correct several anatomical interpretations in the original description. Reassessment of the phylogenetic relationships of *Elachistosuchus* yielded controversial results, but shows that it is a diapsid with possible affinities to the origin of Sauria. We were thus able to reject both Janensch’s [5] and Walker’s [6] hypotheses placing *Elachistosuchus* as a pseudosuchian archosaur and as a rhynchocephalian lepidosaur, respectively. Its possible phylogenetic relationships to morphologically disparate groups such as Choristodera and *Coelurosauravus* reinforce the potential importance of little-known taxa such as *Elachistosuchus* for understanding the early evolutionary history of these groups. The current available conflicting topologies also indicate the inadequacy of our current knowledge of basal saurian phylogeny. Reassessing the anatomy of various poorly known diapsid taxa such as *Elachistosuchus* will help elucidate the relationships of non-saurian neodiapsids and basal archosauromorphs and lepidosauromorphs.

## Supporting Information

S1 File Coding of *Elachistosuchus huenei* for the datasets of Chen *et al*. [12] and Ezcurra *et al*. [13]. (PDF)

S1 Fig "Allcompat" consensus tree from a Bayesian analysis using the character-taxon matrix of Chen *et al*. [12] without the inclusion of *Elachistosuchus huenei*. Numbers indicate the posterior probabilities of branches. Nodes without number indicate a posterior probability of 1.
(TIF)

S2 Fig "Allcompat" consensus tree from a Bayesian analysis using the character-taxon matrix of Ezcurra *et al*. [13] without the inclusion of *Elachistosuchus huenei*. Numbers indicate the posterior probabilities of branches. Nodes without number indicate a posterior probability of 1.
(TIF)

S3 Fig Strict consensus tree of 8 most parsimonious trees, each with a length of 836 steps, from the phylogenetic analysis using the character-taxon matrix of Ezcurra *et al*. [13] with characters unordered and *Elachistosuchus huenei* not included. Numbers above branches indicate bootstrap values. Numbers below branches indicate jackknife values > 50. Bremer support values were lower than 0 for all branches. CI = 0.346 and RI = 0.633.
(TIF)

S4 Fig Strict consensus tree of 4 most parsimonious trees, each with a length of 854 steps, from the phylogenetic analysis using the character-taxon matrix of Ezcurra *et al*. [13] with characters unordered and inclusion of *Elachistosuchus huenei*. Numbers above branches indicate bootstrap values. Numbers below branches indicate jackknife values > 50. Bremer support values were lower than 0 for all branches. CI = 0.338 and RI = 0.630.
(TIF)
