# Supplementary figures and images for: Anatomy of the Enigmatic Reptile Elachistosuchus huenei Janensch, 1949 (Reptilia: Diapsida) from the Upper Triassic of Germany and Its Relevance for the Origin of Sauria
Source: PLoS One. 2015 Sep 9;10(9):e0135114. doi: 10.1371/journal.pone.0135114 (PMC4564268; doi:10.1371/journal.pone.0135114)

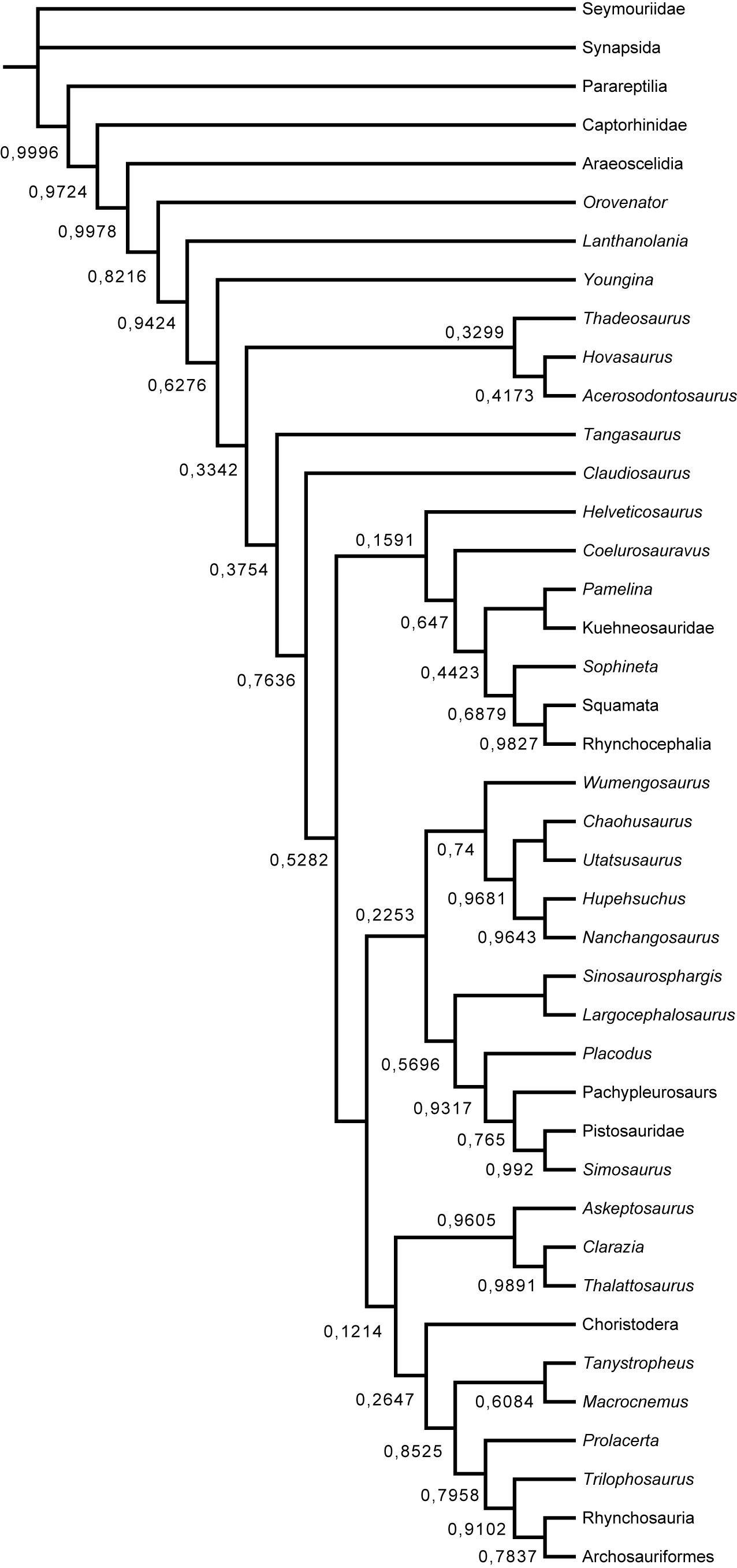

Supplement: S1 Fig — Numbers indicate the posterior probabilities of branches. Nodes without number indicate a posterior probability of 1. (TIF) [file pone.0135114.s002.tif]

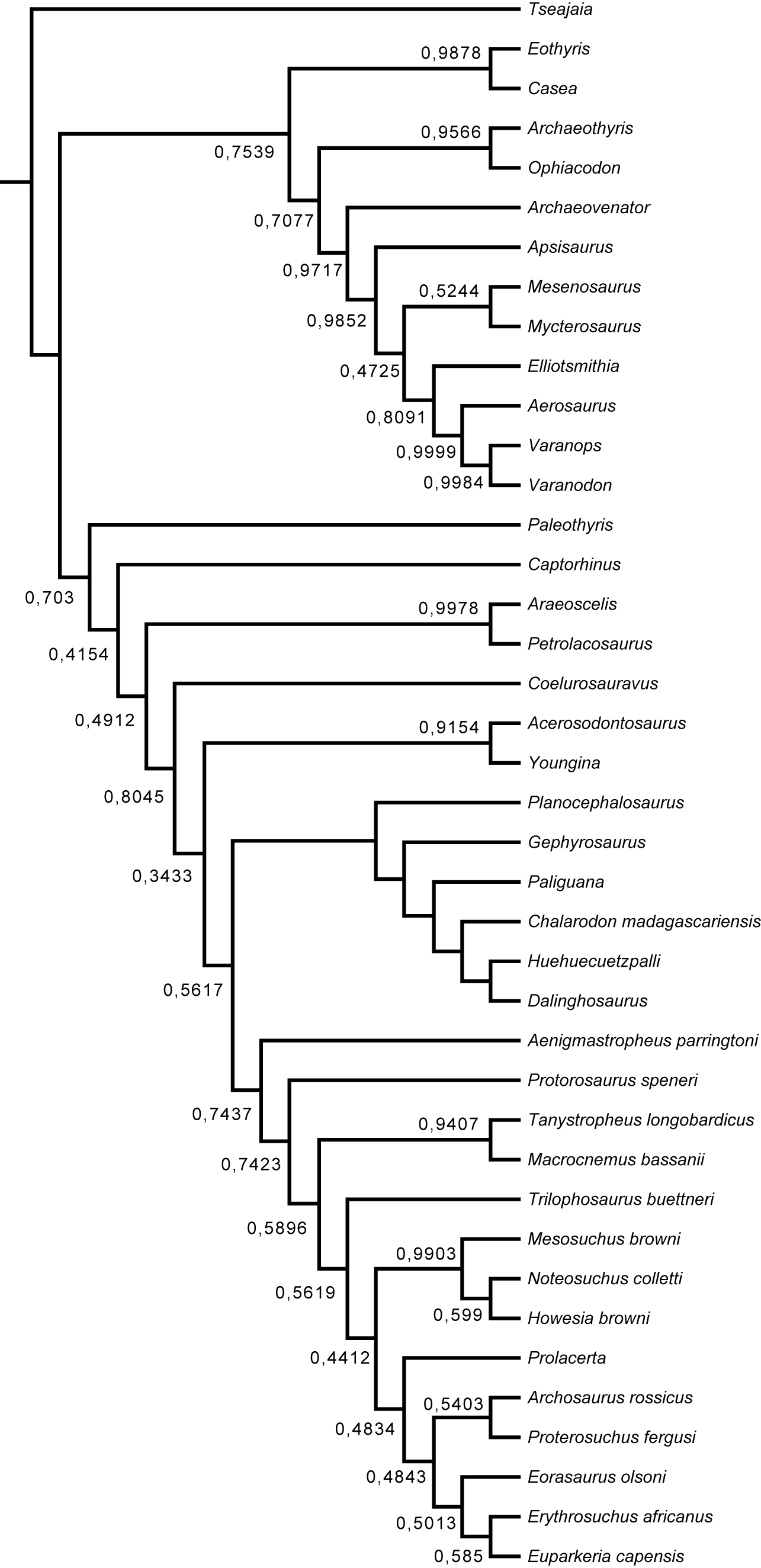

Supplement: S2 Fig — Numbers indicate the posterior probabilities of branches. Nodes without number indicate a posterior probability of 1. (TIF) [file pone.0135114.s003.tif]

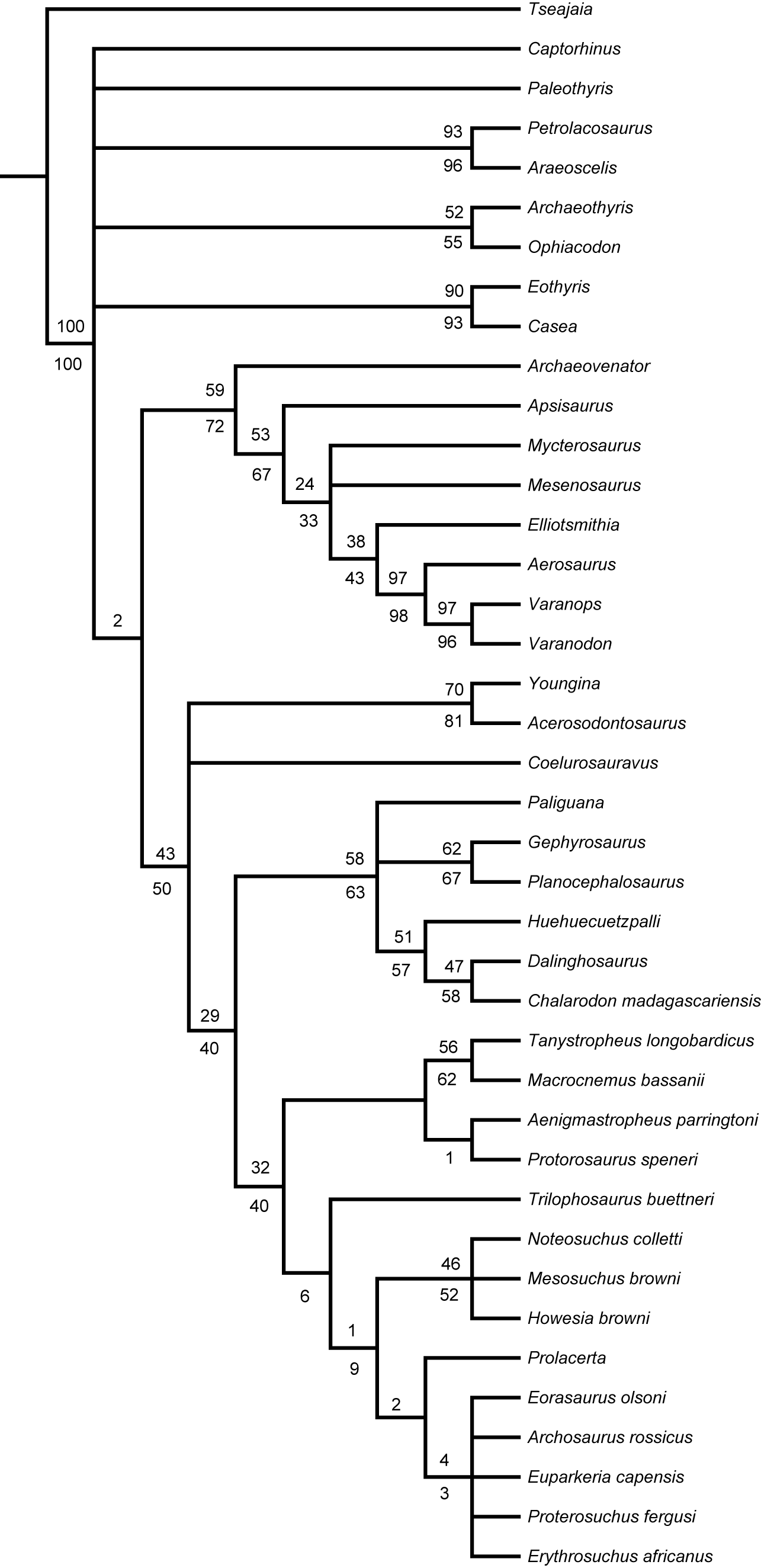

Supplement: S3 Fig — Numbers above branches indicate bootstrap values. Numbers below branches indicate jackknife values > 50. Bremer support values were lower than 0 for all branches. CI = 0.346 and RI = 0.633. (TIF) [file pone.0135114.s004.tif]

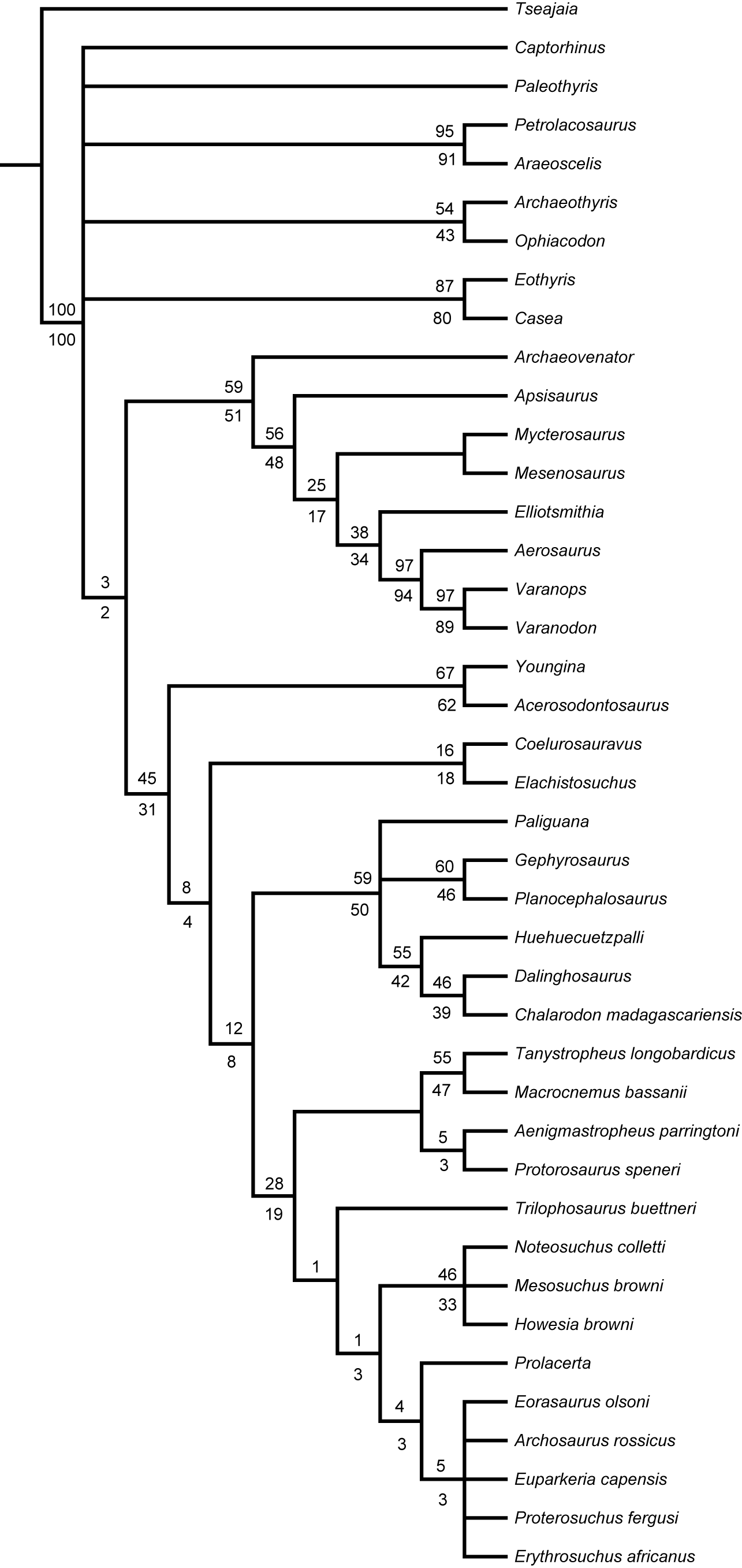

Supplement: S4 Fig — Numbers above branches indicate bootstrap values. Numbers below branches indicate jackknife values > 50. Bremer support values were lower than 0 for all branches. CI = 0.338 and RI = 0.630. (TIF) [file pone.0135114.s005.tif]
