# Supplementary material for: Anatomy of the Enigmatic Reptile Elachistosuchus huenei Janensch, 1949 (Reptilia: Diapsida) from the Upper Triassic of Germany and Its Relevance for the Origin of Sauria
Source: PLoS One. 2015 Sep 9;10(9):e0135114. doi: 10.1371/journal.pone.0135114 (PMC4564268; doi:10.1371/journal.pone.0135114)
Supplement: S1 File — (PDF) [file pone.0135114.s001.pdf]

Chen et al. 2014

Elachistosuchus

?	?	0	?	0	?	0	0	1&2&3	1	0	?
?	1	1	0	2	?	?	?	?	0	1	0
0	?	?	1	?	1	1	0	?	0	1	1
1	0	1	1	1	1	1	0	0	0	0	?
?	?	?	0	0	?	1	0	?	?	?	0
0	?	0	?	?	?	?	?	?	?	?	?
?	?	?	?	?	?	?	?	0	?	?	?
?	?	?	?	?	?	1	1	0	?	0	1
1	?	0	0	0	1	1	0	?	?	?	?
?	?	1	1	?	?	?	?	0	1	1	?
0	0	?	?	0	1	0	1	?	1	?	?
?	?	0	1	1	0	1	0	1	1	?	?
0	?	?	?	?	?	?	?	0	1	1	?
?	0	0	?	0	0	0	1	0	0	0	0
?	?	?	?	?	?	?	?	1	?	?	?
?	?	1	?	?	?	?	?	?	?	?	?
?	0&2	0	?	0	?	0	0	0	1	?	0
1	?	?	?	0	?	?	?	?			

Ezcurra et al. 2014

Elachistosuchus

0	2	0	0	0	0	0	?	?	?	?	?
1	1	1	0	-	?	?	?	?	?	?	2
1	2	1	?	0	?	?	?	0	?	?	?
?	1&2	1&2	3	0	?	?	?	?	?	1	2
0	0	2	2	2	?	?	0	0&1	?	1	1
1	1	0	?	?	1	1	?	0	0	?	?
1	1	0	0	?	0	?	0	1	0	0	?
1	?	?	0	1	?	?	?	?	?	?	?
?	?	?	?	?	?	?	?	?	?	?	?
?	?	?	?	0	?	?	?	?	0	-	?
0	0	1	0	0	0	?	2	1	?	?	?
0	1	1	0	0	1	2	0	1	0	0	?
0	0	0	?	?	?	1	1	0	?	?	?
?	2	0	0	0	?	0	?	?	0	0	1
?	?	?	?	1	0	1	1	?	0	0	0
0	0	0	0	0	0	?	?	?	?	?	?
0	0	1	1	0	?	?	?	?	?	?	?
?	?	?	?	?	?	?	?	?	?	?	?
?	?	?									
